# Genome-wide identification, characterization and expression analysis of the BMP family associated with beak-like teeth in *Oplegnathus*

**DOI:** 10.3389/fgene.2022.938473

**Published:** 2022-07-18

**Authors:** Yuting Ma, Yongshuang Xiao, Zhizhong Xiao, Yanduo Wu, Haixia Zhao, Guang Gao, Lele Wu, Tao Wang, Ning Zhao, Jun Li

**Affiliations:** ^1^ School of Marine Science and Engineering, Qingdao Agricultural University, Qingdao, China; ^2^ CAS and Shandong Province Key Laboratory of Experimental Marine Biology, Center for Ocean Mega-Science, Institute of Oceanology, Chinese Academy of Sciences, Qingdao, China; ^3^ Southern Marine Science and Engineering Guangdong Laboratory (Guangzhou), Guangzhou, China; ^4^ Laboratory for Marine Biology and Biotechnology, Qingdao National Laboratory for Marine Science and Technology, Qingdao, China; ^5^ College of Marine Science, University of Chinese Academy of Sciences, Beijing, China; ^6^ Weihai Haohuigan Marine Biotechnology Co., Weihai, China

**Keywords:** *Oplegnathus fasciatus*, *Oplegnathus punctatus*, tooth development, BMP, gene family, adaptive evolution, gene expression

## Abstract

Bone morphogenetic proteins (BMPs), which belong to the transforming growth factor beta (TGF-β) family, are critical for the control of developmental processes such as dorsal-ventral axis formation, somite and tooth formation, skeletal development, and limb formation. Despite *Oplegnathus* having typical healing beak-like teeth and tooth development showing a trend from discrete to healing, the potential role of BMPs in the development of the beak-like teeth is incompletely understood. In the present study, 19 and 16 BMP genes were found in *O. fasciatus* and *O. punctatus*, respectively, and divided into the BMP2/4/16, BMP5/6/7/8, BMP9/10, BMP12/13/14, BMP3/15 and BMP11 subfamilies. Similar TGFb and TGF_β gene domains and conserved protein motifs were found in the same subfamily; furthermore, two common tandem repeat genes (BMP9 and BMP3a-1) were identified in both *Oplegnathus fasciatus* and *Oplegnathus punctatus*. Selection pressure analysis revealed 13 amino acid sites in the transmembrane region of BMP3, BMP7, and BMP9 proteins of *O. fasciatus* and *O. punctatus*, which may be related to the diversity and functional differentiation of genes within the BMP family. The qPCR-based developmental/temporal expression patterns of BMPs showed a trend of high expression at 30 days past hatching (dph), which exactly corresponds to the ossification period of the bones and beak-like teeth in *Oplegnathus*. Tissue-specific expression was found for the BMP4 gene, which was upregulated in the epithelial and mesenchymal tissues of the beak-like teeth, suggesting that it also plays a regulatory role in the development of the beak-like teeth in *O. punctatus*. Our investigation not only provides a scientific basis for comprehensively understanding the BMP gene family but also helps screen the key genes responsible for beak-like tooth healing in *O. punctatus* and sheds light on the developmental regulatory mechanism.

## Introduction

Bone morphogenetic proteins (BMPs) are potent growth factors forming the largest subfamily of the transforming growth factor beta (TGF-β) superfamily. The first BMP was isolated from demineralized bone tissue and named for its ability to induce ectopic endochondral osteogenesis after transplantation into rodent soft tissue (Wozney et al., 1988). To date, over 30 members have been identified in humans, with varying functions during processes such as embryogenesis, skeletal formation, haematopoiesis and neurogenesis (Ducy and Karsenty, 2000; Bragdon et al., 2011). Based on the structural homology and known functions of BMPs, they can typically be classified into five subgroups: the BMP2/4, BMP5/6/7/8, BMP9/10, and BMP12/13/14 groups (von Bubnoff and Cho, 2001; Mazerbourg and Hsueh, 2006). Unlike other BMP family members, BMP1 has a unique protein domain and is not a TGF-β superfamily member but a shrimp hubstopene family member (Bond and Beynon, 1995; Xie et al., 2020). However, studies in zebrafish have shown the importance of BMP1 for bone formation and stability (Asharani et al., 2012). The expression patterns of the BMP gene family and its roles in the bone metabolism and skeletal development of vertebrates have been reported. BMP-2 functions to regulate early dorsal-ventral patterning in vertebrate embryonic development (Rafael et al., 2006). BMP2/4/16 family members participate in various physiological processes during early embryonic development and are highly expressed in scale tissue. However, BMP16 is present in teleost fish but not in other tetrapods (Marques et al., 2016). BMP4 is secreted and synthesized by osteoblasts and can induce the differentiation and proliferation of undifferentiated mesenchymal cells and the formation of cartilage and new bone (Cheng et al., 2003; Canalis, 2009). BMP3 inhibits osteocyte differentiation and can negatively modulate bone density (Daluiski et al., 2001). Another report showed that BMP3 promotes the proliferation of mesenchymal stem cells through the TGF-β signaling pathway (Zhou et al., 2015) BMP9 has been reported to be involved in liver physiology and pathology (Desroches-Castan et al., 2019). For example, it promotes the proliferation, survival, invasion and cancer stem cell properties of hepatocellular carcinoma (HCC) cells (Li et al., 2013). BMP10 is essential for maintaining cardiac growth during murine cardiogenesis (Chen et al., 2004). BMP15 is an oocyte-specific growth factor that is specifically essential for female fertility. It co-regulates folliculogenesis and the ovulation rate with GDF9 (Galloway et al., 2000; Patino et al., 2017). BMP11 (also called growth differentiation factor 11, GDF11) is closely related to GDF-8 (myostatin) (Geng et al., 2010). BMP11 plays a critical role in regulating the axial skeleton in murine bone morphogenesis as well as promoting the formation of mesoderm and neural tissue (Gamer et al., 1999; McPherron et al., 1999). BMP-14 (also known as growth differentiation factor 5, GDF5) plays a crucial role in inducing the formation of ectopic cartilage (Hotten et al., 1996). Additional studies have shown that GDF5 can promote bone formation, cartilage formation, and longitudinal bone growth in the extremities (Tsumaki et al., 1999). BMP14 (also known as GDF5), BMP13 (also known as GDF6) and BMP12 (also known as GDF7) play important roles in the repair and regeneration of tendon/ligament injuries in rats (Wolfman et al., 1997). Studies have shown that BMP5 induces cartilage formation, and soft tissue defects in mice arise from a lack of BMP5 signaling in the subepithelial mesenchyme (King et al., 1994). BMP6 and BMP7 are involved in the formation and compartmentalization of the mid-heart cushion during mouse development (Kim et al., 2001). Moreover, BMP6 and BMP7 also play important roles in kidney diseases (Dendooven et al., 2011; Goncalves and Zeller, 2011). Studies have shown that BMP-8 may be an important player in bone metabolism, particularly in the response to glucocorticoids (Kosa et al., 2011).

The striped knifejaw, *Oplegnathus fasciatus*, and the spotted knifejaw, *Oplegnathus punctatus*, the latter of which is well known for its high nutritional and economic value, are economically important marine fishes in China (Bai et al., 2021). Due to its economic importance, *Oplegnathus* has become the subject of extensive research in various fields such as ecology, physiology, nutrition and evolutionary genomics (Xiao et al., 2020; Gong et al., 2022a; Gong et al., 2022b). *O. fasciatus* and *O. punctatus* inhabit rocky and coral reef areas offshore. They are carnivorous fishes with sharp teeth that allow them to bite through hard shells such as those of shellfish or sea urchins. Both *O. fasciatus* and *O. punctatus* have a distinctive parrot-like beak formed by the fusion of small teeth and tooth germs on the lower jaw (Kakizawa et al., 1980). All species of the *Oplegnathus* family have these highly specialized beak-like teeth, which help them catch and chew hard-bodied prey, such as decapods, echinoderms, and molluscs. *Oplegnathus woodwardi* consumes a substantial volume of sponges, which is facilitated by its possession of beak-like teeth (Maschette et al., 2020). The teeth of *Oplegnathus* heal with the upper and lower jaws to form strong beak-like teeth. Because of their strong teeth and biting ability, *Oplegnathus* fishes have also been called the “king of iso fishing” and are among the most challenging and interesting fishes to catch. Members of the teleost order *Tetraodontiformes*, such as *Triodontidae* (three-toothed pufferfishes), *Molidae* (ocean sunfishes), *Diodontidae* (porcupinefishes), and *Tetraodontidae* (four-toothed pufferfishes), have also evolved a variety of morphologically distinct beak-like teeth (Andreucci et al., 1982), and these unique and diverse teeth facilitate wide dietary niche occupancy, including access to hard-shelled prey (Turingan, 1994). BMPs, especially BMP2, BMP4, and BMP7, are essential for tooth development, not only inducing the proliferation of mesenchymal cells in the initial process of tooth germ development and participating in the construction of developmental signalling centres but also functioning as a basic protein for the terminal differentiation of ameloblasts and odontoblasts (Liu and Lian, 2018; Liu and Lian, 2018). To further explore the role of BMPs in the development of beak-like teeth, genome-wide identification and annotation of BMP genes in *Oplegnathus* were carried out in this study. In addition, the genetic structure and evolutionary characteristics of these genes as well as their expression characteristics in different tissues and different developmental stages were also analysed. First, from an ecological point of view, the BMP gene family is the key regulator of beak-like tooth development, and the BMP/Smads pathway is involved in the regulation of osteoblast differentiation and bone formation. For example, the BMP2, BMP4 and BMP7 genes are essential for tooth development. Moreover, Oplegnathidae fishes have special healing beak-like teeth, so we were interested in investigating the regulatory roles of BMPs in beak-like tooth development and the healing process. Second, at the evolutionary level, we selected several species of fish with and without healing teeth and some representative higher animals as references to investigate potential differences in BMP family composition through evolutionary analysis.

## Materials and methods

### Ethics statement

All experiments strictly abided by the experimental animal guidance policy of the experimental animal ethics committee of the Institute of Oceanology, Chinese Academy of Sciences (Permit Number: IOCAS2022012).

### Experimental materials

*O. punctatus* were obtained from the Fish Breeding Base of The Institute of Oceanology, Chinese Academy of Sciences (Haihe Aquatic Seedling Co., Ltd., Weihai Wendeng). To detect the differential expression of BMP genes among the tissues of *O. punctatus*, we anaesthetized three adult *O. punctatus* with MS-222, dissected and collected eight different tissues (gills, liver, spleen, brain, the surrounding tissue of the beak-like teeth, intestine, muscle, and heart), and preserved the tissue samples in liquid nitrogen for further analysis.

### Morphological observation of the beak-like teeth of *Oplegnathus*

Three-year-old *Oplegnathus* samples were selected, their teeth were dissected, and the surface structure of the beak-like teeth was observed under a ZEISS Stemi 2000-c stereoscope. Otherwise, samples were imaged with high-resolution, high-contrast scans by a ZEISS Xradia 515 Versa 3D X-ray microscope at 20.0 and 3.0 µm voxel resolutions. Afterwards, ZEISS 3D Viewer software was used to observe the reconstructed 3D structure of the beak-like teeth of *Oplegnathus*, and the teeth were rendered in 3D using Dragonfly software. Samples of *O. punctatus* juveniles were collected for skeletal staining observation. Bone staining was performed according to the methods described by Dingerkus and Uhler (1977). Samples were stored in glycerol after processing, and a small amount of thymol was added to prevent bacterial contamination. Bone-stained *O. punctatus* samples were placed in a Petri dish with glycerol, observed and photographed with a ZEISS Stemi 2000-c stereoscope.

### Identification and sequence analysis of bone morphogenetic protein genes

All available BMP gene sequences and BMP amino acid sequences of nine species (*Cyprinus carpio*, *Salmo salar*, *Danio rerio*, *Oryzias latipes*, *Xenopus laevis*, *Takifugu rubripes*, *Gallus gallus*, *Mus musculus*, and *Homo sapiens*) were downloaded from the NCBI (https://blast.ncbi.nlm.nih.gov/Blast.cgi) and Ensembl (http://asia.ensembl.org/) public databases. A simple HMM search in TBtools software and NCBI Blast were used to search the reference genomes of *O. fasciatus* and *O. punctatus* and preliminarily obtain candidate BMP gene sequences of these two species (Chen et al., 2020). Meanwhile, the NCBI-CDD database (https://www.ncbi.nlm.nih.gov/cdd/) was used to verify the identified characteristic domains of BMP genes in *O. fasciatus* and *O. punctatus*. The SMART (http://smart.embl-heidelberg.de) website was used to predict the structural information of BMP protein domains, such as TGFb and TGF_β. The mass fractions and isoelectric points of the BMP proteins were estimated by the ExPASy online tool (https://web.expasy.org/compute_pi). The reference genome accession codes used for analysis in this study are as follows: *O. fasciatus* (NCBI accession code: PRJNA393383) and *O. punctatus* (CNGB accession code: CNP0001488) (Xiao et al., 2019; Li et al., 2021).

### Phylogenetic analysis and chromosomal locations of bone morphogenetic protein genes

The BMP protein sequences of *O. fasciatus*, *O. punctatus* and the other nine species (*C. carpio*, *S. salar*, *D. rerio*, *O. latipes*, *X. laevis*, *T. rubripes*, *G. gallus*, *M. musculus*, and *H. sapiens*) were aligned using MUSCLE of TBtools (Edgar, 2004). Then, the created alignment file was used for phylogenetic analysis with MEGA11 software to construct a neighbour-joining (NJ) tree and a maximum likelihood (ML) tree (1,000 bootstraps) for comparison (Tamura et al., 2021). The online software EvolView (https://evolgenius.info//evolview-v2/) was used to beautify the evolutionary tree. Multiple sequence alignment of *O. fasciatus* was performed using Jalview software. MEME (http://meme-suite.org/tools/meme) online software was used to analyse BMP motifs. Then, the results were visualized with TBtools software. The conserved domains of BMPs in *Oplegnathus* were obtained using CDD of the NCBI and visualized by TBtools. Chromosome position and gene density information were obtained through the *Oplegnathus* genome database, the BMP gene positions were marked on the chromosomes, the collinear relationships between different chromosomes were obtained through the genome database, and the results were visualized using TBtools software.

### Evolutionary selection pressure analysis of bone morphogenetic protein genes

Phylogenetic trees were constructed individually for BMP gene families from 11 species using the NJ method in MEGA11. Using the CODEML program of PAML4.9j (Yang, 2007), selection pressure analysis was performed on the main clades using different models (branch model, site model and branch-site model). First, branch models are used to determine whether the selection pressure on each branch of the evolutionary tree is significant (Yang, 1998). The main branch models are the one-ratio model (M0), in which the ω-values of all branches in the phylogenetic tree are equal; the free-ratio model (M1), in which the ω-values among branches in the phylogenetic tree are unequal; and the two-ratio model (M2), where the ω-values of foreground branches are different from those of background branches. In addition, the site model assumes that different branches of the phylogenetic tree are subject to the same selection pressure but that different amino acid sites experience different selection pressures. The following are the main site models: the one ratio model (M0), assuming that all sites have the same ω value; the near-neutral model (M1a), assuming that only conserved sites (0 < *ω* < 1) and neutral sites (*ω* = 1) are present; the positive selection model (M2a), in which sites with positive selection (*ω* > 1) are added to M1a; the discrete model (M3), assuming a simple discrete distribution trend of ω values for all sites; the beta model (M7), assuming that *ω* belongs to the matrix (0, 1) and follows a beta distribution for all sites; and the beta and ω model (M8), in which another class of ω values is added to M7, which can be obtained computationally and can be set to 1. Comparisons between M1 and M2, M0 and M3, and M7 and M8 are often used for site models, and M1 and M2 are more stable than M7 and M8. Moreover, in the analysis of the branch-site model, we tested for positively selected amino acid sites among the branches through the settings of different foreground branches and compared the null hypothesis of each Model A (MA) with the corresponding alternative hypothesis (Zhang et al., 2005). Then, the ratio ω of non-synonymous to synonymous substitutions (dN/dS) was calculated. When *ω* = 1, selection is neutral (that is, there is no selection); when *ω* > 1, there is positive selection; and when 0 < *ω* < 1, there is negative selection, also called purifying selection. In this paper, the likelihood ratio test (LRT) was used to compare model pairs, and the Bayesian method (BEB) was used to identify the sites with positive selection (Bielawski and Yang, 2003).

### Expression of bone morphogenetic protein genes

RT–PCR was performed with the CFX96 real-time detection system (Bio-Rad, United States) to determine BMP expression differences in different developmental stages and tissues of *O. punctatus*. Total RNA was isolated from samples of eight different tissues (gill, liver, spleen, brain, the surrounding tissue of the beak-like teeth, intestine, muscle, and heart) and five developmental stages (5 days past hatching (dph), 16, 30, 50, and 60 dph) in *O. punctatus* using TRIzol (Invitrogen) according to the manufacturer’s instructions. Considering that the fish were too small to precisely separate the surrounding tissue of the beak-like teeth, the sampling locations for the five developmental stages were on the head, except for days 5 and 16 dph, when whole fish were taken because they were too small to precisely separate the head. RNA concentration and purity were measured using an ultra-micro spectrophotometer (NanoDrop 2000, United States). In general, the OD260 nm ratio was OD280 nm ≥ 1.8, and the OD260 nm ratio was OD230 nm ≥ 1. RT-PCR was conducted to synthesize cDNA from 1 μg of total RNA by using an Evo M-MLV RT Kit (Accurate Biology, Changsha, China) following the manufacturer’s instructions. The internal positive control was the β-actin gene from *O. punctatus*, with the forward primer (5′-GCT​GTG​CTG​TCC​CTG​TA-3′) and reverse primer (5′-GAG​TAG​CCA​CGC​TCT​GTC-3′). Details of the BMP gene primer pairs are provided in Supplementary Table S1. qRT–PCR experiments were performed with a 20 μl mixture consisting of 0.4 μl of each primer, 10 μl of SYBR^®^ Premix Ex Taq™ II (Tli RNaseH Plus) (2X), 7.2 μl of ddH_2_O, and 2 μl of template cDNA. The PCR program consisted of an initial denaturation step at 95°C for 30 s and 39 denaturation cycles: denaturation at 95°C for 5 s and annealing at 60°C for 30 s. The temperature depended on the primers. Melting curve analysis was performed at the end of the reaction to demonstrate its specificity. Each experiment was performed in triplicate. β-Actin was used for normalization of all gene expression data (Wang et al., 2020). According to the measured Ct value (the average value of three parallel samples for Ct), the relative expression levels of the BMP genes were calculated by Pfaffl’s method (Pfaffl, 2001). All data obtained in the experiment were analysed by one-way analysis of variance using GraphPad Prism 9 software, and *p* < 0.05 was indicative of a statistically significant difference. Quantitative results were plotted using the R environment and GraphPad Prism 9 software.

## Results

### Morphological observation of *Oplegnathus*

**FIGURE 1:**
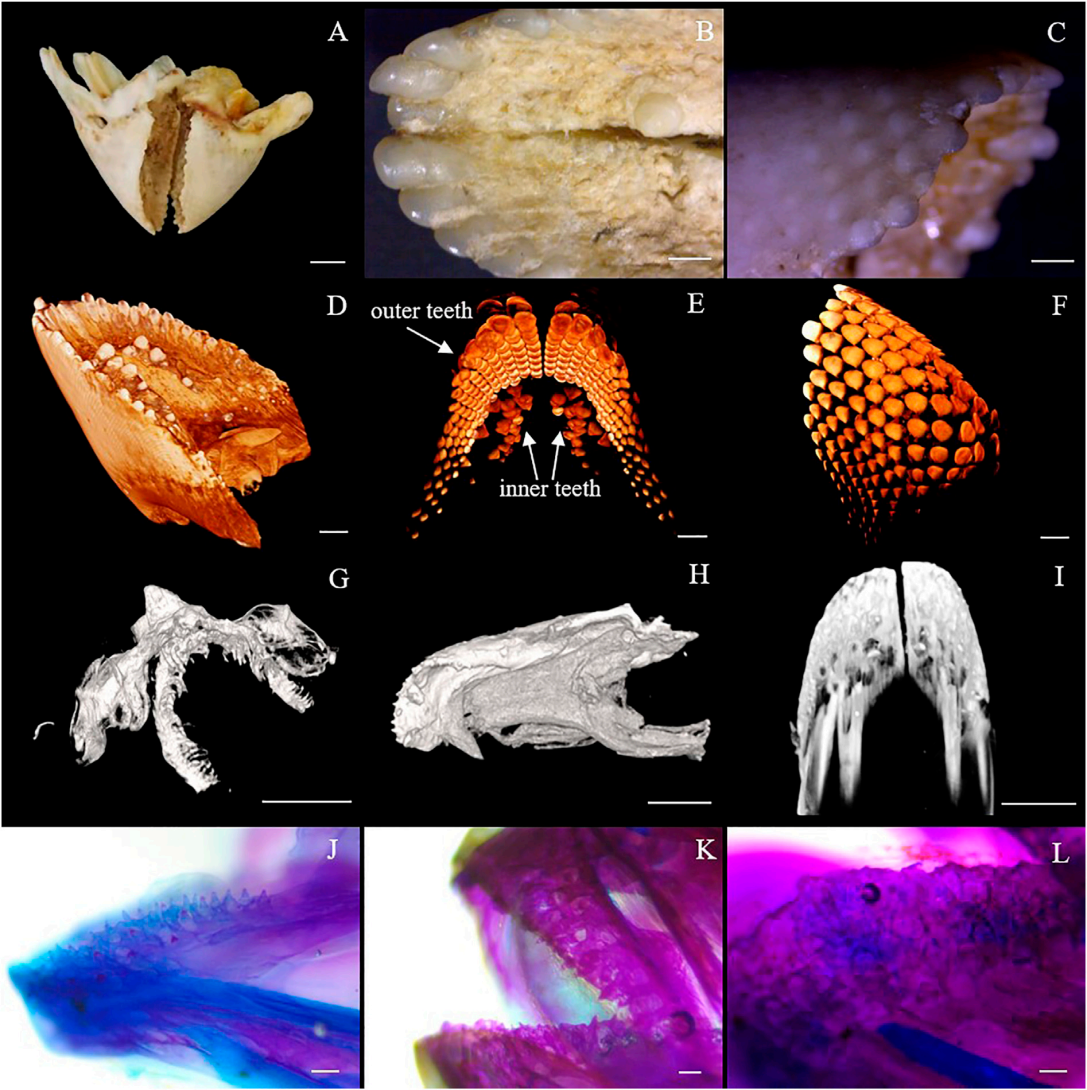
Beak-like tooth morphology of *Oplegnathus*. **(A)** Photographs of the overall morphology of the beak-like teeth of *Oplegnathus*. **(B)** Enlarged image of locoregional details of the inner teeth of *Oplegnathus*. **(C)** Enlarged image of locoregional details of the outer teeth of *Oplegnathus*. **(D–F)** Reconstructed micro-CT scans showing the nested dentition of adult *Oplegnathus*. **(E)** Three-dimensional arrangement of the inner and outer teeth of *Oplegnathus.* **(G–I)** Micro-CT scans showing the developmental changes of the nested dentition of *Oplegnathus* at 30, 50 and 70 dph. **(J–L)** Alizarin red staining results of beak-like teeth in different developmental stages of *Oplegnathus*. **(J)** indicates that the 45-dph beak-like teeth are in a cartilaginous state and separately arranged, **(K)** indicates that the 50-dph beak-like teeth are ossified and have a tendency to heal, and **(L)** indicates that complete healing of the beak-like teeth has occurred by 90 dph (scale bars: 5 mm in **(A)**; 1 mm in **(B,C,I)**; 2 mm in **(D,F)**; 500 μm in **(G,H)**; 100 μm in F and H; and 100 μm in **(J–L)**.

As shown in Figure 1, the *Oplegnathus* species have a unique healing beak-like teeth structure, with the upper and lower jaw teeth showing healing and a gap in the middle. In addition, there are obvious traces of the accumulation of replacement teeth on the outer edges of the beak-like teeth. Moreover, the outer layer of the beak-like teeth is filled with smooth calcium, while the inner layer is relatively rough. As shown in Figure 1, the 3D rendering analysis of the thickness of the teeth by micro-CT revealed a nested internal arrangement of the teeth of the *Oplegnathus* species, extending from far from the rostral end to near the rostral end. In addition, several irregularly distributed internal teeth could be observed on the inner edge of the beak-like teeth (Figure 1E). The micro-CT scan results at 30, 50 and 70 dph showed beak-like teeth distributed in a single row of separation at 30 dph, replacement teeth at 50 dph, beak-like teeth that generally tended to heal, and complete rostral tooth healing at 70 dph (Figures 1G–I). The results of bone staining are shown in Figures 1J–L. At 45 dph, the beak-like teeth are individually arranged conical teeth, and most of the teeth and bones are stained blue with bromophenol blue, showing a cartilaginous state; only the tips of the teeth, which are beginning to ossify, are red. After 50 dph, the discrete arrangement of conical teeth shows a tendency to heal, and all of the beak-like teeth are stained with alizarin red, indicating that ossification has been completed. By 90 dph, the beak-like teeth of *O. punctatus* had healed. External observation showed clear signs of replacement tooth accumulation on the outer edges of the upper and lower teeth, with three to four rows of replacement teeth.

### Bone morphogenetic protein gene family identification

**FIGURE 2:**
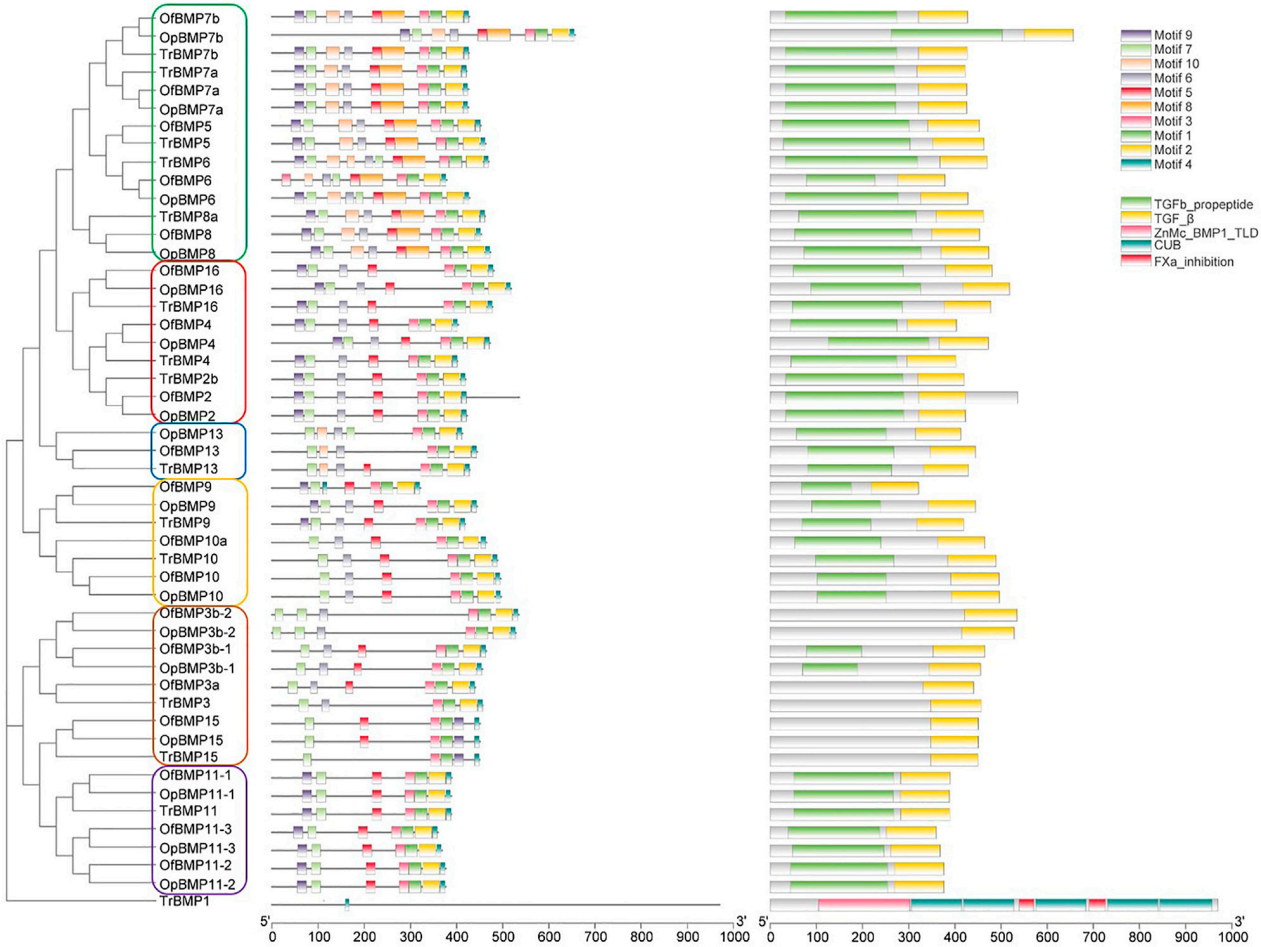
The conserved BMP domains and motifs in *Oplegnathus* and *T. rubripes*.

**FIGURE 3:**
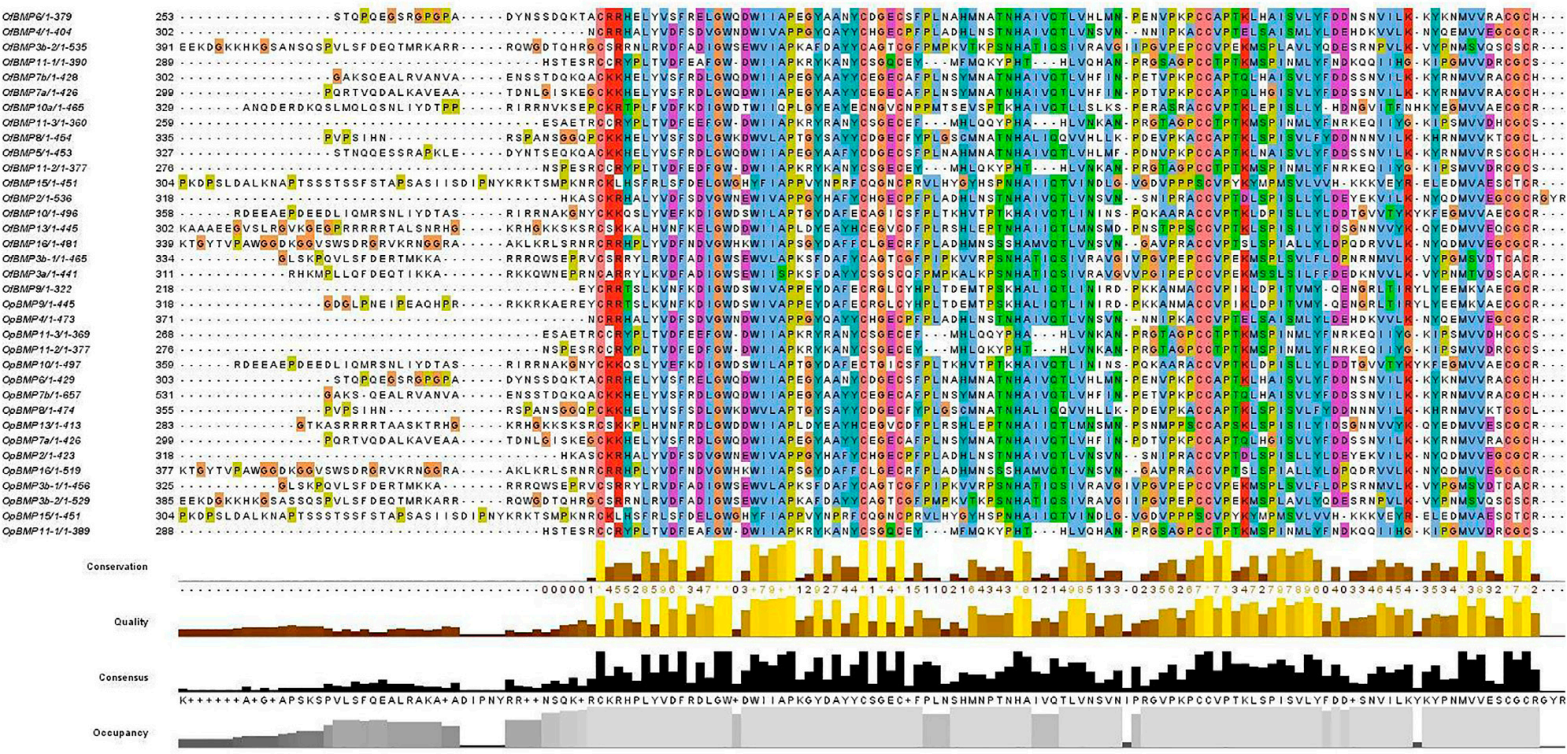
Multiple sequence alignment of the conserved domains of proteins in the *Oplegnathus* BMP gene family.

**FIGURE 4:**
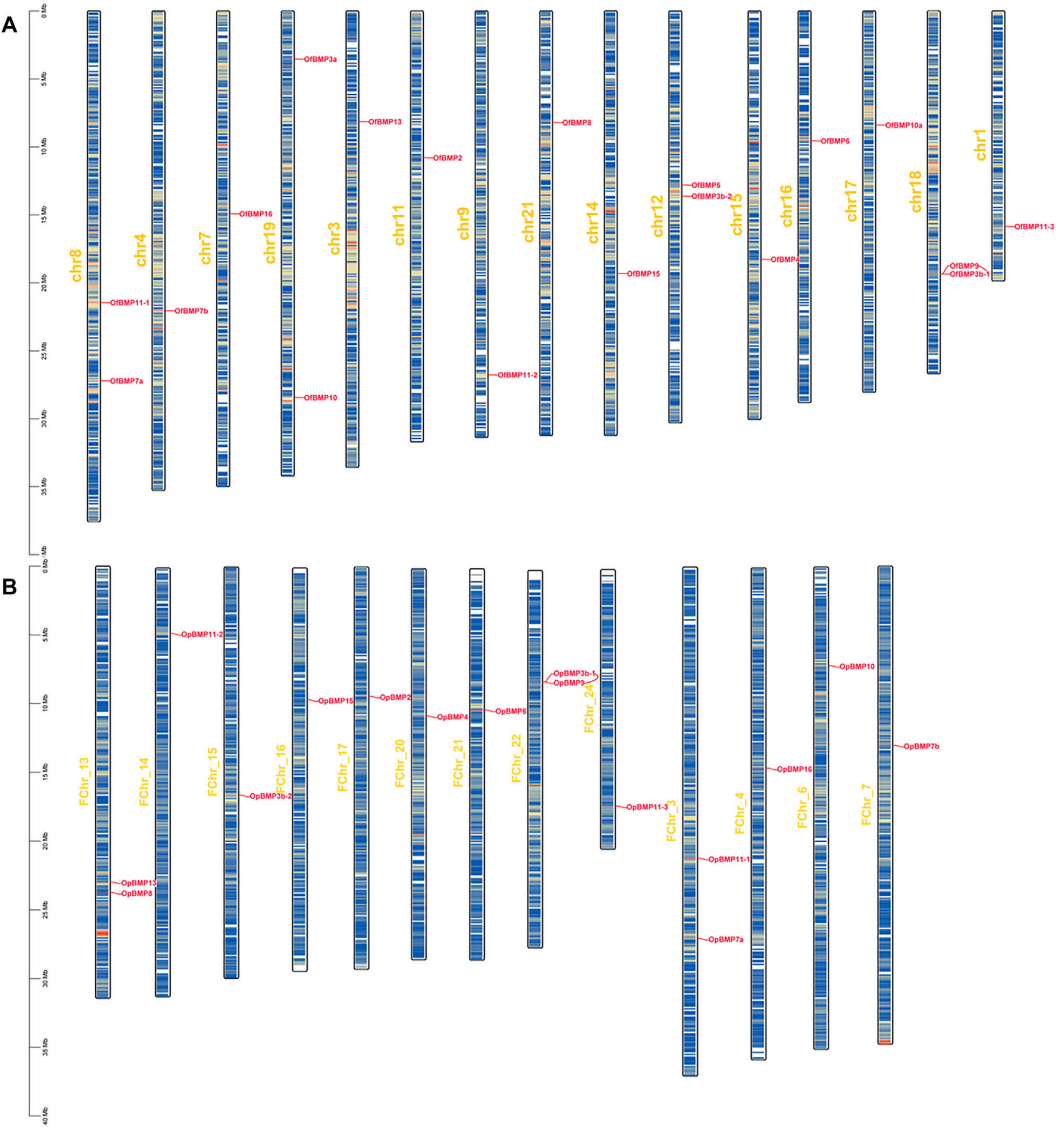
Chromosome localization of BMP genes in *O. fasciatus* and *O. punctatus*. **(A)** Represents *O. fasciatus*; **(B)** represents *O. punctatus*. The prefix “F" in the chromosome names in Panel **(B)** indicates that the reference genomic data are from female *O. punctatus*.

**FIGURE 5:**
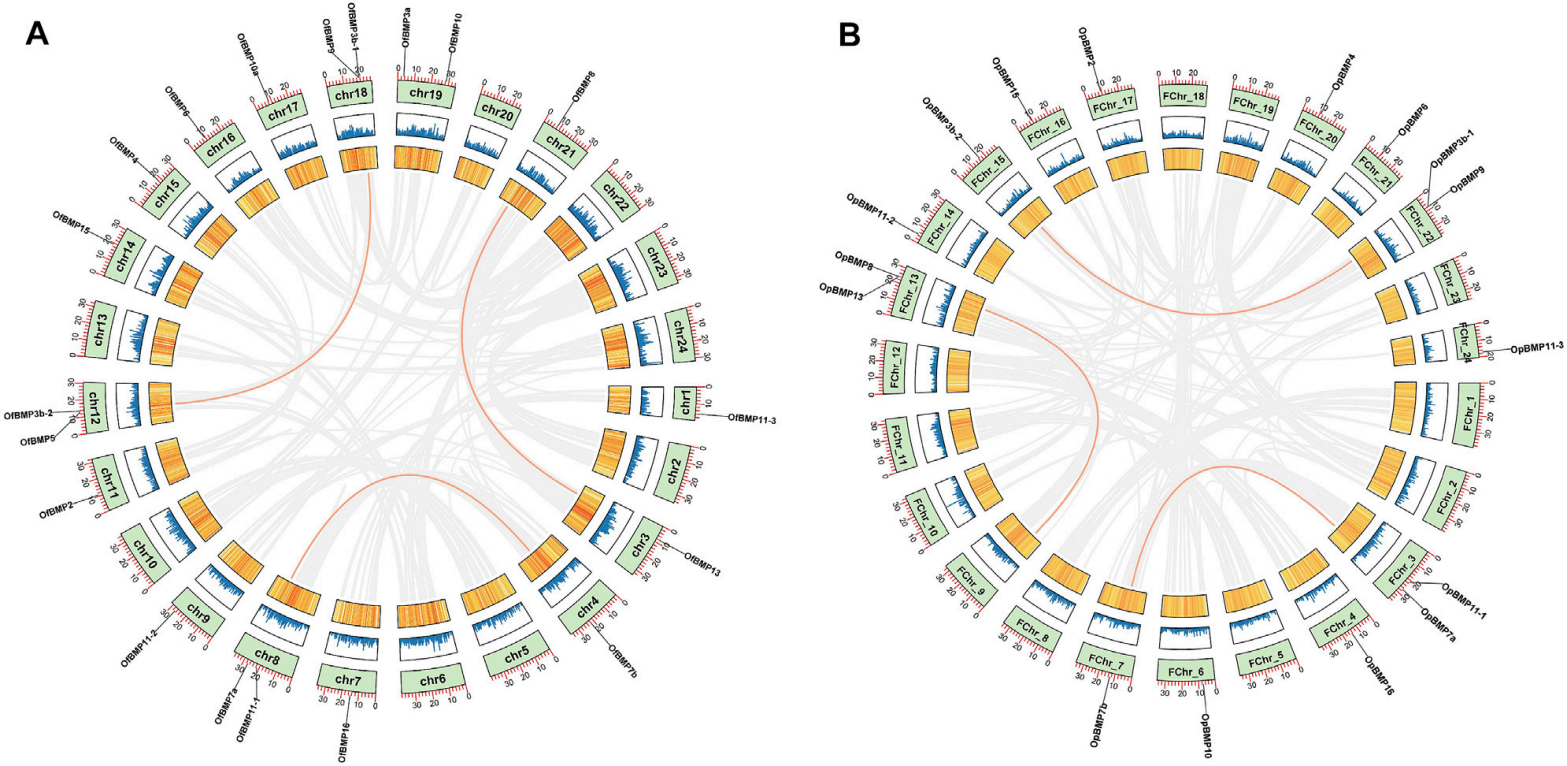
Chromosome localization and collinearity analysis of BMP genes in *O. fasciatus* and *O. punctatus*. **(A)** Represents *O. fasciatus*; **(B)** represents *O. punctatus*.

The cDNA sequence characteristics and accession numbers of all cloned BMP genes of *Oplegnathus* are shown in Table 1. The accession numbers of all cloned BMP genes of the other 11 species are shown in Supplementary Table S2. 19 and 16 different BMP genes were found in the genomes of *O. fasciatus* and *O. punctatus*, respectively (Table 1). In Table 1, genes with the prefix “Of” before “BMP” are BMP genes in *O. fasciatus*, and those with the prefix “Op” are BMP genes in *O. punctatus*. The length of the coding sequence (CDS) is 966–1,971 bp, the length of the encoded protein is 321–656 amino acids, the molecular weight is between 36 and 73 kDa, and the isoelectric point is between 5.09 and 9.72. As shown in Figure 2, to deeply investigate the structural diversity of the BMP gene family, the conserved domains and motifs of the *Oplegnathus* BMP proteins were visualized by TBtools software. Except BMP1, the *Oplegnathus* BMPs have similar TGF_β domains, indicating that the BMP gene family belonging to the TGF-β superfamily is highly conserved. In addition, most BMP genes, except BMP3 and BMP15, also possess a TGFb propeptide domain, revealing their potentially differentiated functions in biological processes. Furthermore, a total of 10 distinct conserved motifs were identified. The conserved domains and motifs were compared according to the phylogenetic relationships between *Oplegnathus* and *T. rubripes* (please refer to Supplementary Figure S1 for a description of the names and lengths of the motifs). The highly consistent conserved domains and motifs in the same subfamilies indicated similar protein functions among members of the same subfamily. To explore the conserved domains and phylogenetic relationships of the BMP proteins of *O. fasciatus* and *O. punctatus*, multiple sequence alignment of their TGF_β domains was performed, revealing that all BMP family members contain a highly conserved TGF_β region at the C-terminus, which consists of approximately 100–110 amino acids (Figure 3). As shown in Figures 4A, 19 BMPs were distributed among 15 of the 24 chromosomes in *O. fasciatus*. Both BMP3b-1 and BMP9 were located on chromosome 10 and had tandem duplications. As shown in Figures 4B, 16 BMPs were distributed among 13 of the 24 chromosomes in *O. fasciatus*. Both BMP3b-1 and BMP9 were located on chromosome 22. As shown in Figure 5A, the homologous fragment of the OfBMP3b-1 gene located on chromosome 18 of *O. fasciatus* is the OfBMP3b-2 gene located on chromosome 12. Moreover, the homologous fragment of the OfBMP7a gene located on chromosome 8 of *O. fasciatus* is the OfBMP7b gene located on chromosome 4. Furthermore, the homologous fragment of OfBMP13 on chromosome 3 of *O. fasciatus* is located on chromosome 21. As observed in *O. fasciatus*, the homologous fragment of the OpBMP3b-1 gene located on chromosome 22 in *O. punctatus* is the OpBMP3b-2 gene located on chromosome 15. Additionally, the homologous fragment of the OpBMP7a gene located on chromosome 3 in *O. punctatus* is the OpBMP7b gene located on chromosome 7. Furthermore, the homologous fragment of OpBMP13 on chromosome 13 of *O. punctatus* is located on chromosome 9 (Figure 5B).

**TABLE 1:** Summary of the characteristics of BMP genes in *O. fasciatus* and *O. punctatus*.

Gene name	Gene ID	Location	CDS (na)	CDS (aa)	PI	MW (Da)	Accession number
OfBMP 2	Opa012474	Chr11	1608	535	6.93	60,430.29	ON881245
OfBMP 3a	Opa007971	Chr19	1323	440	9.6	50,769.68	ON881248
OfBMP 3b-1	Opa022377	Chr18	1395	464	9.32	52,078.46	ON881246
OfBMP 3b-2	Opa018624	Chr12	1605	534	9.3	60,351.16	ON881247
OfBMP 4	Opa019617	Chr15	1212	403	7.74	46,269.59	ON881249
OfBMP 5	Opa018570	Chr12	1359	452	8.96	50,980.73	ON881250
OfBMP 6	Opa020205	Chr16	1137	378	8.73	42,817.76	ON881251
OfBMP 7a	Opa003248	Chr8	1278	425	5.98	48,584.1	ON881252
OfBMP 7b	Opa005368	Chr4	1284	427	6.53	48,861.37	ON881253
OfBMP 8	Opa015260	Chr21	1362	453	9.19	51,095.91	ON881254
OfBMP 9	Opa022376	Chr18	966	321	5.81	36,617.86	ON881255
OfBMP 10a	Opa021035	Chr17	1395	464	6.13	52,883.03	ON881256
OfBMP 10	Opa008866	Chr19	1488	495	5.14	55,413.19	ON881257
OfBMP 11-1	Opa003044	Chr8	1170	389	6.67	44,283.68	ON881258
OfBMP 11-2	Ofa014976	Chr9	1131	376	5.6	42,776.95	ON881259
OfBMP 11-3	Ofa023623	Chr1	1080	359	6.27	40,682.57	ON881260
OfBMP 13	Opa010039	Chr3	1335	444	9.25	50,612.51	ON881261
OfBMP 15	Opa016633	Chr14	1353	450	9.56	51,083.92	ON881262
OfBMP 16	Opa006329	Chr7	1443	480	9.49	53,148.13	ON881263
OpBMP2	mikado.FChr_17G371.1	FChr_17	1269	422	8.59	47,680.8	ON881264
OpBMP3b-1	mikado.FChr_22G218.1	FChr_22	1368	455	9.39	51,047.4	ON881265
OpBMP3b-2	mikado.FChr_15G610.1	FChr_15	1587	528	9.15	59,774.4	ON881266
OpBMP4	mikado.FChr_20G478.1	FChr_20	1419	472	8.38	54,316.1	ON881267
OpBMP6	mikado.FChr_21G268.1	FChr_21	1287	428	6.99	48,845.4	ON881268
OpBMP7a	mikado.FChr_3G1132.1	FChr_3	1278	425	5.98	48,578.1	ON881269
OpBMP7b	mikado.FChr_7G612.1	FChr_7	1971	656	6.4	73,424.9	ON881270
OpBMP8	mikado.FChr_13G995.1	FChr_13	1422	473	9.22	53,168.2	ON881271
OpBMP9	mikado.FChr_22G219.1	FChr_22	1335	444	5.9	50,131.1	ON881272
OpBMP10	mikado.FChr_6G239.1	FChr_6	1491	496	5.09	55,597.3	ON881273
OpBMP11-1	mikado.FChr_3G854.1	FChr_3	1167	388	6.67	44,185.6	ON881274
OpBMP11-2	mikado.FChr_14G143.1	FChr_14	1131	376	5.6	42,777	ON881275
OpBMP11-3	mikado.FChr_24G619.1	FChr_24	1107	368	6.47	41,654.7	ON881276
OpBMP13	mikado.FChr_13G951.1	FChr_13	1239	412	9.16	47,141	ON881277
OpBMP15	mikado.FChr_16G452.1	FChr_16	1353	450	9.45	51,100	ON881278
OpBMP16	mikado.FChr_4G590.1	FChr_4	1557	518	9.72	57,520.1	ON881279

### Phylogenetic analysis

**FIGURE 6:**
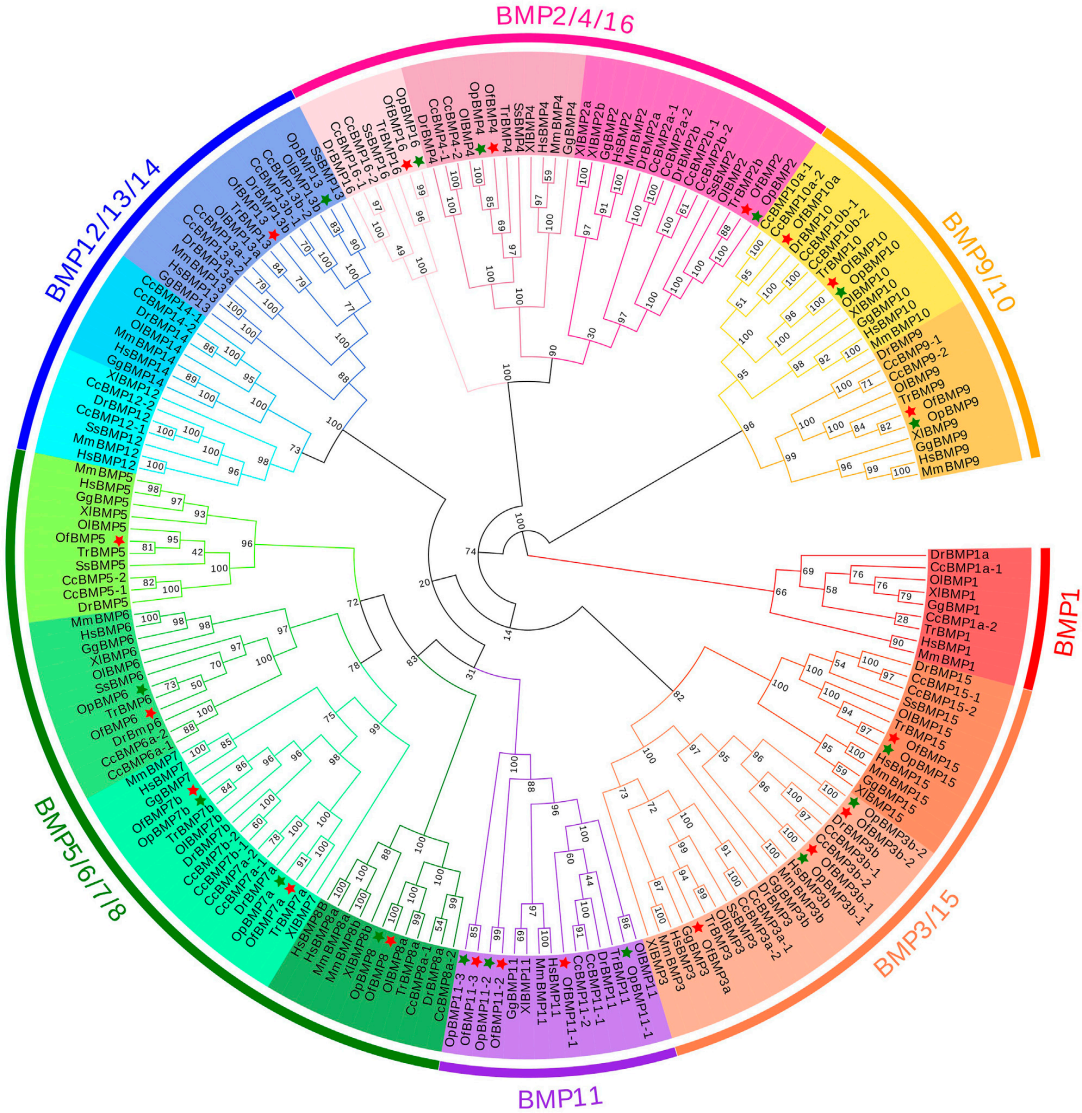
Phylogenetic tree of BMP gene family members in 11 species.

**FIGURE 7:**
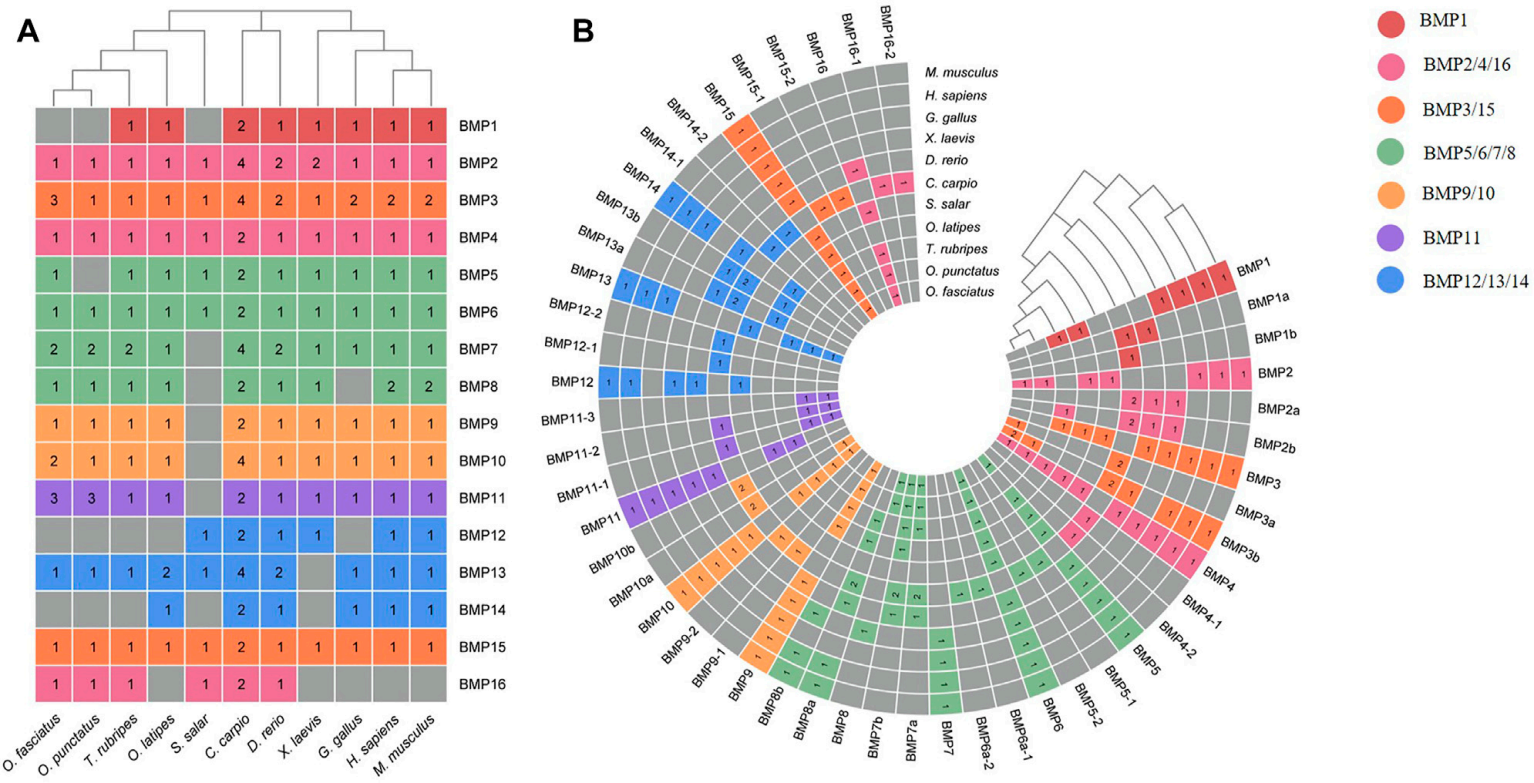
Heatmap of gene copy number comparisons of BMP genes in 11 species. **(A)** Shows a summary of the gene copy number comparison. **(B)** Shows the detailed gene copy number comparison. The different colors in the Figure refer to different BMP subfamilies, the numbers on the heatmap indicate the number of gene copies, and the gray color indicates the deletion of the BMP genes.

To demonstrate the phylogenetic relationships of BMP genes between *Oplegnathus* and other species, we selected a total of 198 BMP amino acid sequences from 11 species, namely, *O. fasciatus*, *O. punctatus, C. carpio, S. salar, D. rerio, O. latipes, X. laevis, T. rubripes, G. gallus, M. musculus,* and *H. sapiens*. The phylogenetic tree was constructed by the ML method using MEGA11 and TBtools. As shown in Figure 6, the high topological consistency of the phylogenetic relationships of the BMPs indicates that they are highly evolutionarily conserved. Based on structural homology, except for BMP1, the BMPs were divided into six subfamilies, namely, the BMP2/4/16, BMP5/6/7/8, BMP9/10, BMP12/13/14, BMP3/15, and BMP11 groups. Both *O. fasciatus* and *O. punctatus* contain three members of the BMP11 gene family: BMP11-1, BMP11-2, and BMP11-3. *O. fasciatus* has three members of the BMP3 gene family: OfBMP3a, OfBMP3b-1, and OfBMP3b-2. *O. punctatus* has two members of the BMP2 gene family: OpBMP3b-1 and OpBMP3b-2. The copy number of each BMP gene in nine vertebrates was summarized and analysed. The details of the numbers of BMP members among the 11 species are provided in Table 2. Except in *C. carpio*, the number of BMP family members in the vertebrates ranges from 14 to 22. *C. carpio* (Xu et al., 2014; Chen et al., 2017) experienced an additional whole-genome duplication event, giving it approximately double the number of paralogous genes in zebrafish. As shown in Figure 7, BMP1, BMP12, and BMP14 were not found in *Oplegnathus* or *T. rubripes*. Additionally, BMP16 has been found only in fish species. BMP13 and 14 are absent in *X. laevis,* while BMP8 and BMP12 are absent in *G. gallus*.

**TABLE 2:** Comparison of copy numbers of BMP genes among selected vertebrate genomes.

	*O. fasciatus*	*O. punctatus*	*C. carpio*	*S. salar*	*D. rerio*	*O. latipes*	*T. rubripes*	*X. laevis*	*G. gallus*	*M. musculus*	*H. sapiens*
BMP1			2		1	1	1	1	1	1	1
BMP2	1	1	4	1	2	1	1	2	1	1	1
BMP3	3	2	4	1	2	1	1	1	2	2	2
BMP4	1	1	2	1	1	1	1	1	1	1	1
BMP5	1		2	1	1	1	1	1	1	1	1
BMP6	1	1	2	1	1	1	1	1	1	1	1
BMP7	2	2	4		2	1	2	1	1	1	1
BMP8	1	1	2		1	1	1	1		2	2
BMP9	1	1	2		1	1	1	1	1	1	1
BMP10	2	1	4		1	1	1	1	1	1	1
BMP11	3	3	2		1	1	1	1	1	1	1
BMP12			2	1	1			1		1	1
BMP13	1	1	4	1	2	2	1		1	1	1
BMP14			2		1	1			1	1	1
BMP15	1	1	2	1	1	1	1	1	1	1	1
BMP16	1	1	2	1	1		1				
Total	19	16	42	9	20	15	15	14	14	17	17

### Evolutionary selection pressure analysis of bone morphogenetic protein genes

In the branch-site model, we set the branch on which the target BMP genes of *O. fasciatus* and *O. punctatus* were located as the foreground branch, and the branch on which the BMP genes of the other species were located was set as the background branch. According to the results in Table 3, we identified three genes (BMP3, BMP7, and BMP9) with positively selected sites under the branch-site model. In contrast, no positively selected sites were detected for BMPs in the branch model and site model, and the omega values were less than 1, indicating that the BMP genes are strongly negatively selected under this model. In the branch-site model, for the BMP3 gene, the following 11 amino acid sites were positively selected: 136H, 138K, 140V, 141F, 143F, 145L, 146S, 148I, 150E, 151S, and 153L. Similarly, 559T of BMP7 and 26F of BMP9 were identified as positively selected sites. Furthermore, the LRTs show that the above results are reliable, and the alternative hypothesis Model A is accepted. The specific parameters and results of the model are shown in Supplementary Tables S4–S9. Please refer to the Supplementary Material for details on the model parameters and LRTs for other BMP genes.

**TABLE 3:** Likelihood ratio test statistics for BMP3, BMP7 and BMP9.

	Model	Model comparison	Positive selection site (BEB)	df	LRT (2ΔL)	*p*	Accepted model
	Branch model	M0 vs. M1		36	97.986196	0.00000012	M1
		M0 vs. M2-3a		1	6.545132	0.01051734	M2
		M0 vs. M2-3b		1	0.012826	0.90992187	M0
	Site model	M0 vs. M3		4	1113.924544	0	M0
		M1a vs. M2a		2	0	1.00000000	M1a
		M7 vs. M8		2	2.606358	0.27167467	M7
		MA vs. null-3a	136 H 0.978*				
			138 K 0.993**				
BMP3			140 V 0.994**				
			141 F 0.987*				
			143 F 0.956*				
	Branch-site model		145 L 0.971*	1	56.971496	0.00000000	MA
			146 S 0.998**				
			148 I 0.996**				
			150 E 0.988*				
			151 S 0.998**				
			153 L 0.995**				
		MA vs. null-3b		1	7.507388	0.00616990	MA
	Branch model	M0 vs. M1		30	133.166076	0.00000000	M1
		M0 vs. M2-7a		3	11.228174	0.01055428	M2
		M0 vs. M2-7b		3	1.817434	0.61115584	M0
BMP7	Site model	M0 vs. M3		4	569.175784	0	M3
		M1a vs. M2a		2	0	1.00000000	M1a
		M7 vs. M8		2	1.294466	0.52350955	M7
	Branch-site model	MA vs. null-7a	559 T 0.959*	1	12.347524	0.00044158	MA
		MA vs. null-7b		1	0	1.00000000	Null
	Branch model	M0 vs. M1		18	67.531604	0.00000012	M1
		M0 vs. M2		1	4.203038	0.04035253	M2
BMP9	Site model	M0 vs. M3		4	697.961866	0	M3
		M1a vs. M2a		2	0	1.00000000	M2a
	Branch-site model	M7 vs. M8		2	19.615106	0.00005504	M8
		MA vs. null	26 F 0.996**	1	22.64134	0.00000195	MA

*(*p* < 0.05); **(*p* < 0.01)

### Tissue-specific expression of bone morphogenetic protein genes in *Oplegnathus punctatus*

**FIGURE 8:**
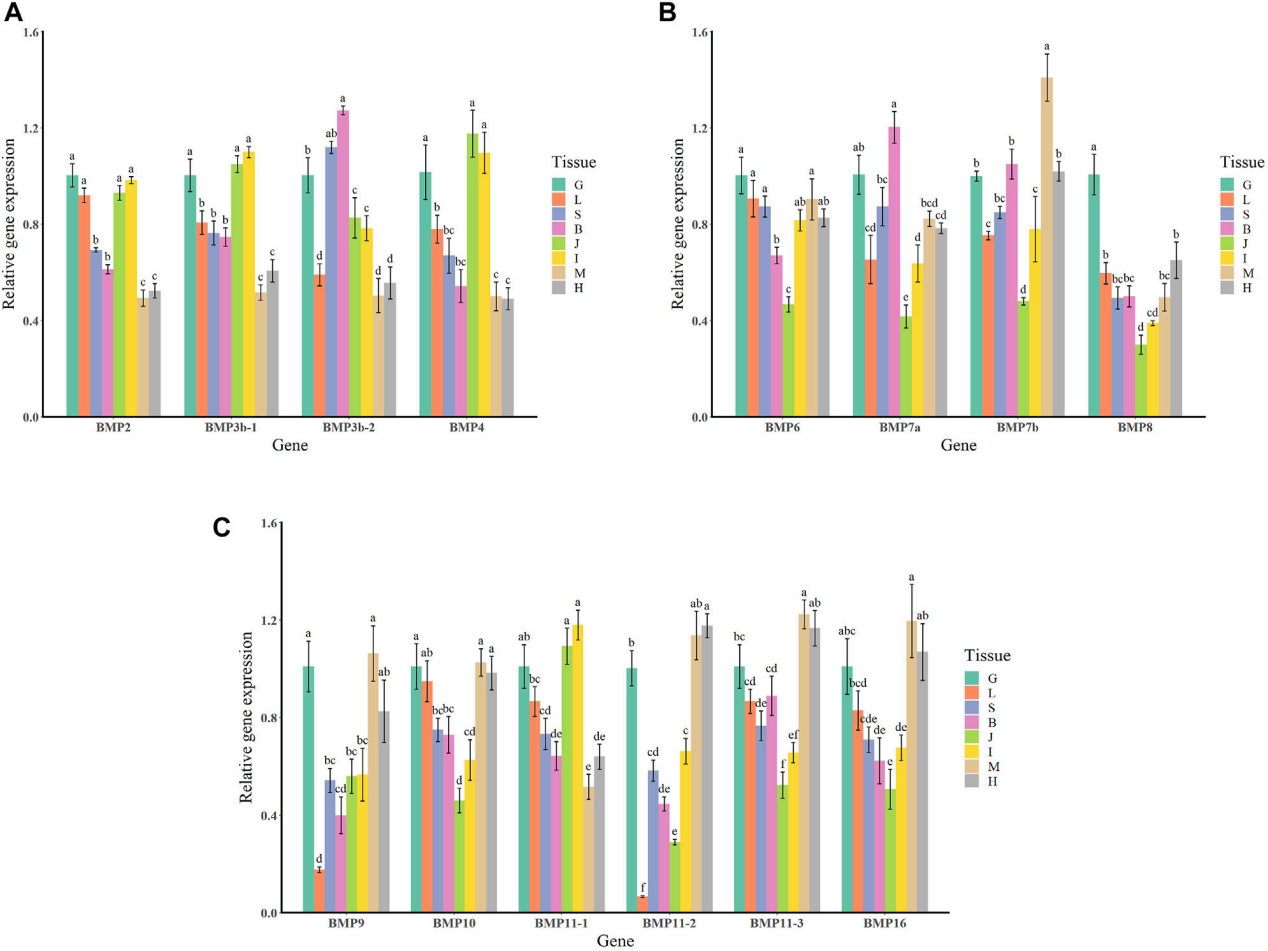
Relative expression levels of BMP genes in selected tissues of *O. punctatus*. **(A)** Shows the tissue-specific expression patterns of BMP2, BMP3b-1, BMP3b-2 and BMP4; **(B)** shows the tissue-specific expression patterns of BMP6, BMP7a, BMP7b and BMP8; **(C)** shows the tissue-specific expression patterns of BMP9, BMP10, BMP11-1, BMP11-2, BMP11-3 and BMP16. G represents the gills; L represents the liver; S represents the spleen; B represents the brain; J represents the surrounding tissue of the beak-like teeth; I represents the intestine; M represents the muscle; H represents the heart. Different lowercase letters above the standard error bars indicate significant differences in expression.

The qRT–PCR expression results showed that the BMP genes were expressed in various healthy tissues of *O. punctatus*, but the expression patterns of these genes varied among the tissues. The gill expression values were selected as a reference for comparison with the expression values of other tissues during the analysis of PCR gene expression data, so the relative expression value for gill tissue was set at 1. In general, most BMP genes were widely expressed, and there was a trend for higher relative expression of most BMP genes in the gill, as this tissue is more likely to be associated with BMP-related pathways. As shown in Figure 8, regarding our focus on the epithelial tissue and mesenchymal tissue of the beak-like teeth, the high relative expression of BMP2, BMP3b-1, BMP3b-2, BMP4, and BMP11-1 in the epithelial tissue and mesenchymal tissue of the beak-like teeth (all values above 1) is interesting. However, the relative expression of BMP3b-2 in the spleen and brain was higher than that in the epithelial tissue and mesenchymal tissue of the beak-like teeth. The relative expression of the remaining BMP genes in the epithelial tissue and mesenchymal tissue of the beak-like teeth was low (all below 0.6). Based on the tissue expression patterns of BMP genes, several BMP genes (BMP2, BMP3b-1, BMP3b-2, BMP4, and BMP11-1) that were highly expressed in the surrounding tissue of the beak-like teeth were screened. As shown in Figure 10, their developmental expression patterns at 30, 50, and 60 dph were also analyzed in correlation with the results of alizarin red staining at early developmental stages. As shown in Figures 8A–C, there are obvious differences in the expression levels of the BMP2/4/16 subfamily. The expression abundance of BMP2 was higher in the gills, liver, intestine and the epithelial tissue and mesenchymal tissue of the beak-like teeth, followed by the spleen and brain, with lower levels in the muscle and heart. BMP3b-1 and BMP4 exhibited similar expression patterns; however, their expression in the liver was significantly lower than that of BMP2. In contrast, BMP16 was highly expressed in the muscle, heart and gill, while the lowest expression was detected in the epithelial tissue and mesenchymal tissue of the beak-like teeth. Unlike BMP3b-1, BMP3b-2 is most highly expressed in the brain, followed by the spleen and gill, and weakly expressed in the muscle and heart. As shown in Figure 8B, the BMP5/6/7/8 subfamily exhibited various expression levels across the examined tissues. There was significant downregulation in the epithelial tissue and mesenchymal tissue of the beak-like teeth for the BMP6, BMP7a, BMP7b, and BMP8 genes. The expression of BMP6 and BMP8 was the highest in the gill. BMP7a was highly expressed in the brain, while BMP7b was highly expressed in the muscle. As shown in Figure 8C, the BMP9/10 subfamily showed higher expression in the gill, muscle and heart. For BMP9, the expression level was the lowest in the liver, followed by the brain, spleen, the surrounding tissues of beak-like teeth, and intestine. The expression of BMP10 was higher in the liver, followed by the spleen, brain and intestine, and the lowest expression was observed in the surrounding tissues of the beak-like teeth. As shown in Figure 8C, members of the BMP11 subfamily were mainly expressed in the gill. Additionally, BMP11-1 was also highly expressed in the intestine and the surrounding tissues of the beak-like teeth, while BMP11-2 and BMP11-3 were highly expressed in the muscle and heart. Furthermore, there was significant downregulation in the liver for BMP11-2.

### Temporal expression patterns of bone morphogenetic protein genes in *Omobranchus punctatus*

**FIGURE 9:**
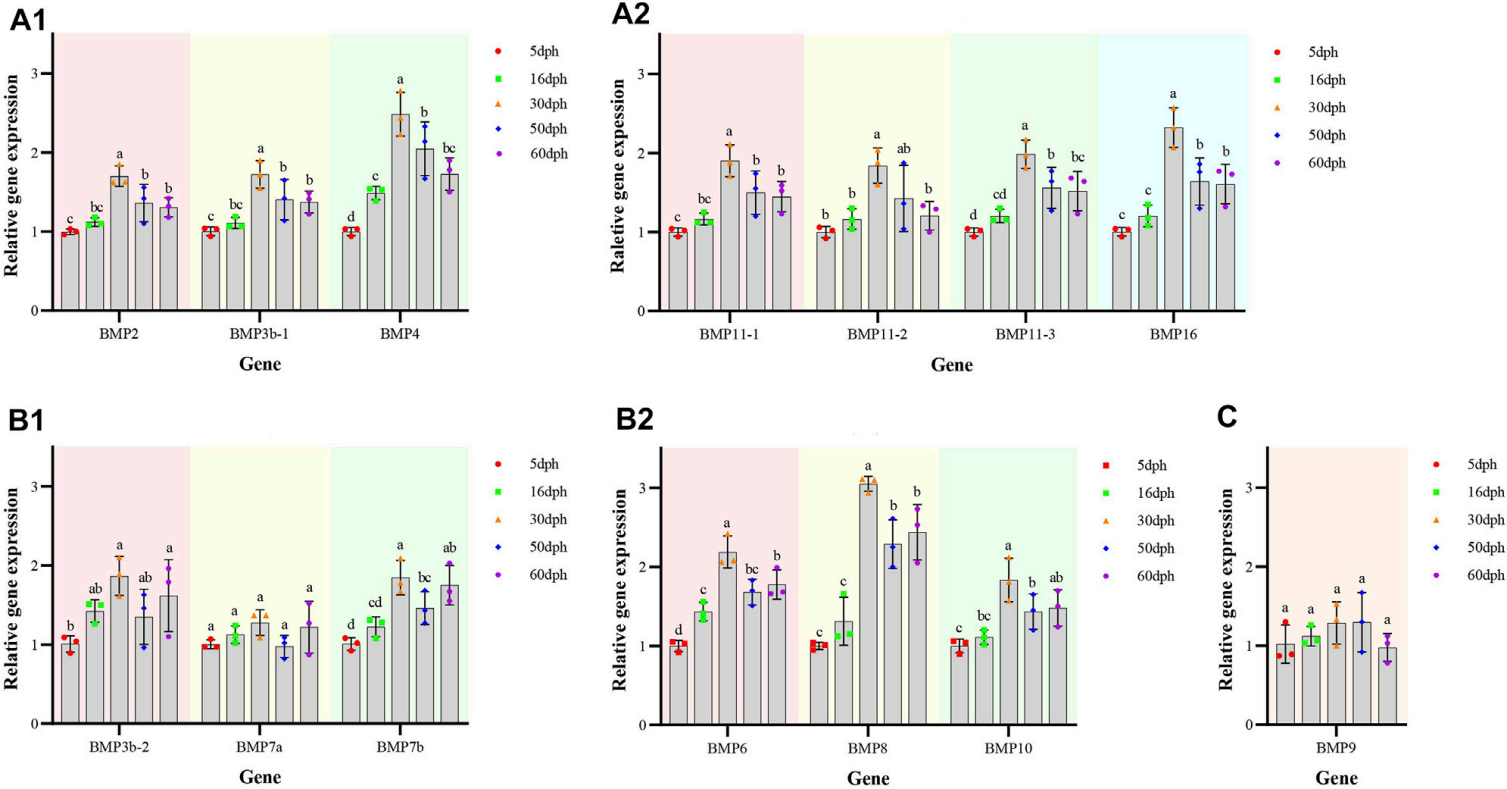
Relative expression levels of BMP genes in selected developmental stages of *O. punctatus*. **(A1,A2)** Represent temporal expression pattern one; **(A1)** shows the temporal expression pattern of BMP2, BMP3b-1 and BMP4; **(A2)** shows the temporal expression pattern of BMP11-1, BMP11-2, BMP11-3, and BMP16. **(B1,B2)** Represent temporal expression pattern two; **(B1)** shows the temporal expression pattern of BMP3b-2, BMP7a and BMP7b; **(B2)** shows the temporal expression pattern of BMP6, BMP8 and BMP10. **(C)** Represents temporal expression pattern three, the temporal expression pattern of BMP9 ***.***

**FIGURE 10:**
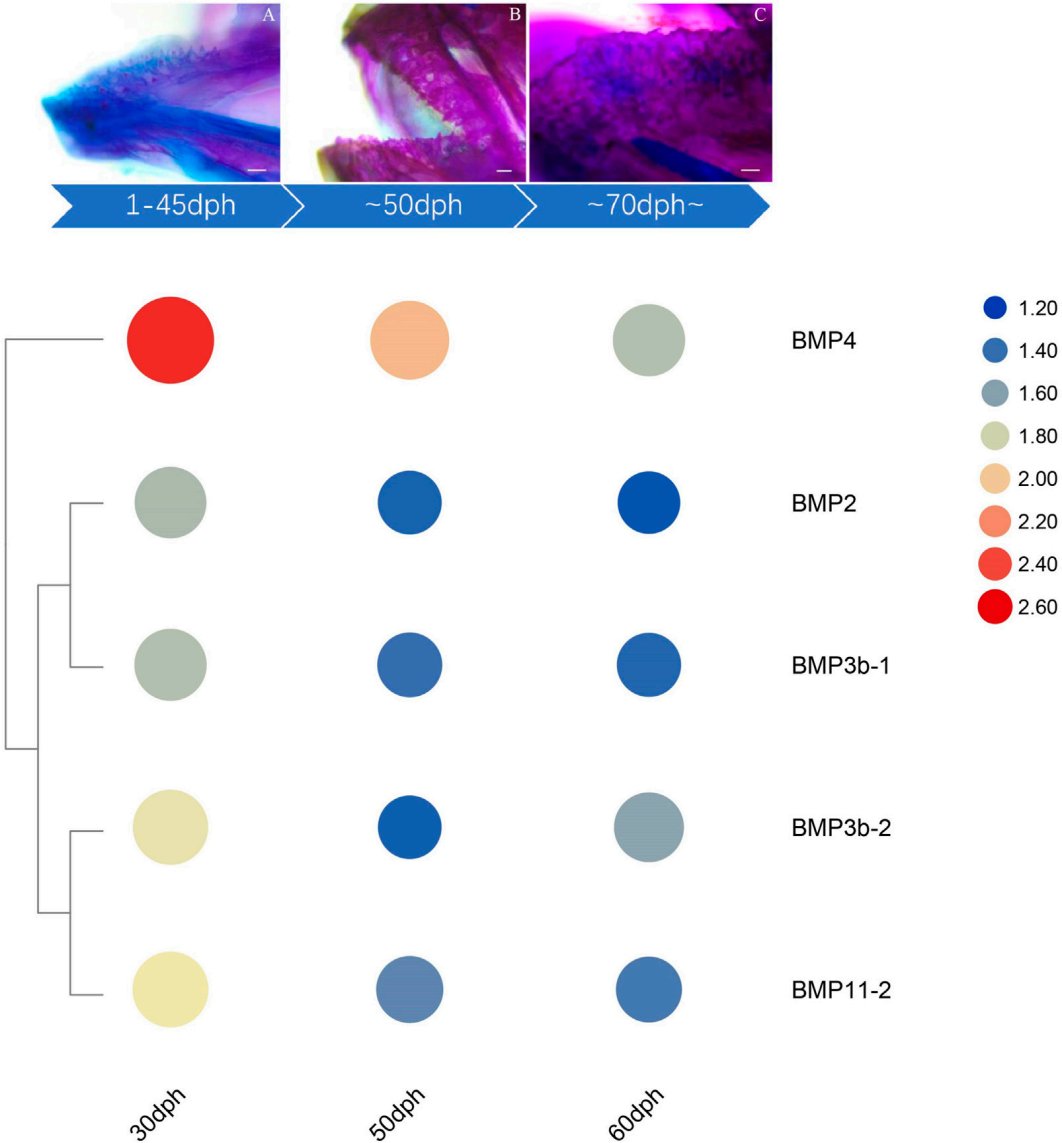
Expression of beak-like teeth-specific BMP genes at critical nodes in beak-like teeth development of *O. punctatus*. The upper panel indicates the results of alizarin red bone staining at critical time of beak-like teeth development, and the lower panel of heatmap indicates the chronological expression of BMP genes that are highly expressed in the surrounding tissue of the beak-like teeth.

The temporal expression patterns of BMP genes during different developmental stages of *O. punctatus* were analyzed by real-time qRT–PCR. The mRNA abundances of different BMP genes in five developmental stages were compared. BMP8 was the most abundantly expressed BMP gene during *O. punctatus* development. Notably, the relative expression levels of all BMP genes were significantly upregulated from 5 to 30 dph and peaked at 30 dph, with the exception of BMP9. Moreover, the lowest relative expression of all genes was observed at 5 dph. As shown in Figure 10, most of the BMP genes, especially BMP2, BMP3b-1, BMP3b-2, BMP4, and BMP11-1, were abundantly expressed at 30 dph, when the beak-like teeth were conical teeth that had not yet healed and only the tips of the teeth were ossified. The expression of BMPs significantly increased during the period from 16 to 30 dph, which indicates that osteoblasts are rapidly generated at this stage and that activation of ossification in *O. punctatus* occurs in preparation for the sclerosing of bones and beak-like teeth. The expression of BMP genes across development can be divided into three patterns. BMP2, BMP3b-1, BMP4, BMP11-1, BMP11-2, BMP11-3, and BMP16 exhibited the first expression pattern (Figure 9A): the expression levels increased from 5 to 30 ph, with the highest expression at 30 dph, and decreased sequentially from 30 to 60 dph, but the relative expression at 60 dph was still higher than the starting value (5 dph). In addition, the relative mRNA expression levels of BMP2, BMP3b-1, BMP11-1, BMP11-2, and BMP11-3 showed similar trends at the different developmental stages of *O. punctatus*. However, the relative mRNA expression levels of BMP4 and BMP16 were significantly higher than those of the above five genes. The second expression pattern (Figure 9B) was different from the first, with the expression levels of BMP3b-2, BMP6, BMP7a, BMP7b, BMP8, and BMP10 increasing from 50 to 60 dph. Compared with other BMP genes, BMP7a showed no significant change in mRNA abundance from 5 to 60 dph. In addition, BMP8 showed a significant increase in mRNA expression at 30–60 dph. For the third expression pattern (Figure 9C), there was a continuous and subtle trend of increasing BMP9 mRNA expression from 5 to 50 dph, with the expression decreasing to a minimum at 60 dph.

## Discussion

To deeply explore the physiological role of *Oplegnathus* BMPs in jaw formation and development, systematic homology comparison was performed to identify the sequences of BMPs and determine their expression patterns. Phylogenetic analysis showed that the BMP11 subfamily of *O. fasciatus* and *O. punctatus* contained three members, BMP11-1, BMP11-2, and BMP11-3. The clustering results for BMP11-1 were consistent with the classification results of other species, while BMP11-2 and BMP11-3 clustered individually. According to the tissue-specific expression pattern of the BMP11 subfamily in *O. punctatus*, BMP11-2 was similar to BMP11-3, and its expression was upregulated in heart and muscle tissues. In contrast, BMP11-1 was downregulated in heart and muscle tissues and upregulated in intestinal tissue and the epithelial tissue and mesenchymal tissue of the beak-like teeth. Findings in mice suggest that BMP11 specifies positional identity along the anterior/posterior axis (McPherron et al., 1999). According to the clustering results, it is speculated that the BMP11-1 in these fishes functions similarly to that in mice and plays an important role in the development of the skeleton and teeth. This also explains the upregulated expression in the tissues around the beak-like teeth of *O. punctatus* shown in Figure 8. In contrast, the downregulated expression of BMP11-2 and BMP11-3 in the surrounding tissues of beak-like teeth suggested weak roles in the formation of the beak-like teeth of *O. punctatus*. The functions of BMP11-2 and BMP11-3 need to be further explored.

The expression changes of target genes during the healing and ossification of *O. punctatus* beak-like teeth were monitored by qRT–PCR. Notably, with the exception of BMP9, the expression of which was not obvious, all BMP genes tested were upregulated at 30 dph. It is inferred that osteoblasts are synthesized rapidly at approximately 30 days of age and enter the period of rapid ossification. For evidence of ossification, we can refer to the study of ossification in juvenile turbot. The parasphenoid bone of the cranial element is the first to undergo ossification beginning at 19 dph in juvenile turbot. Subsequently, maxilla, premaxilla, dentary, frontal, opercular, and preopercular ossification begin. Ossification of the intracranial skeletal system was visible when the juveniles reached full length at 25 dph. The degree of ossification was more obvious at 35 dph (Lv, 2018). As shown in Figure 10, according to the results of alizarin red bone staining, *O. punctatus* had a tapered distribution of teeth at 45 dph, and most of the teeth were stained blue with bromophenol blue, showing a cartilaginous state; only the tips of the teeth were red, indicating that ossification was initiated at the tips of the teeth. Similar findings were reported in pufferfish (Thiery et al., 2017). After 50 dph, discretely arranged tapered teeth showed a tendency to heal, and the teeth were red, indicating that ossification was already complete. This suggests that ossification is a biological outcome that requires the accumulation of osteoblasts before it can occur, and the BMP/Smads pathway is involved in osteoblast differentiation (Ding et al., 2021), while the RT–PCR results showed that most of the BMP genes, especially BMP2, BMP3b-1, BMP3b-2, BMP4, and BMP11-1, were abundantly expressed at 30 dph, presumably preparing the beak-like teeth for healing and sclerosis at 45–50 dph. Compared with that of other genes, the expression of BMP4, BMP8, and BMP16 maintained high levels throughout the five developmental stages of *O. punctatus*, suggesting that they all participate in beak-like tooth growth. Studies have proven that BMP4 can be expressed in the upper beak mesenchyme of Darwin’s finches and is strongly correlated with the regulation of bird beak shape. With increased BMP4 expression, the birds can grow deeper and longer beaks (Abzhanov et al., 2004; Campas et al., 2010). In addition, similar findings have been reported in fish, where the BMP4 gene was involved in the regulation of mandarin fish jaw remodeling (Cao et al., 2020). Studies have also shown that allelic variation and expression of BMP4 are closely related to cichlid jaw morphogenesis. BMP4 may be the main factor responsible for the diversity of jaws in different vertebrate classes (birds and fishes) (Albertson and Kocher, 2006). Based on the upregulated expression of BMP4 in five different developmental stages of *O. punctatus*, this gene may also play a regulatory role in the development of its beak-like teeth. This also explains the high expression of the BMP4 gene in the epithelial tissue and mesenchymal tissue of the beak-like teeth shown in Figure 8. Thus, BMP4 represents a candidate gene for the adaptive evolution of beak-like tooth healing in *O. punctatus*. Studies in zebrafish show that BMP signaling is activated during caudal fin regeneration, and BMP8a expression is upregulated after caudal fin amputation (Schebesta et al., 2006). On the basis of our results that the expression of BMP8 is upregulated at 30 dph and the total expression level is higher than that of other genes, we infer that BMP8 plays an important role in the induction of bone and tooth formation.

Phylogenetic analysis revealed three BMP3 members in *O. fasciatus*: OfBMP3a, OfBMP3b-1, and OfBMP3b-2. *O. punctatus* contains two BMP3 members: OpBMP3b-1 and OpBMP3b-2. The OfBMP3a of *O. fasciatus* was selected as the foreground branch, and the BMP3s of the other species were selected as the background branch for positive selection pressure analysis. The results revealed 11 positively selected amino acid sites. In addition, the expression of BMP3b was upregulated in the epithelial tissue and mesenchymal tissue of the beak-like teeth, as shown in Figure 8. In a study of canine tooth root development, it was shown that BMP3 plays a role in the development of the tooth germ and tooth root and participates in the differentiation of dental follicle cells into cementoblasts and periodontal cells (Xuan et al., 2006). However, most studies suggest that BMP3 functions as an antagonist of BMPs, while BMP3 synthesis is generally dependent on BMPs, suggesting that a feedback mechanism is required to maintain the balance between BMPs and their antagonists. This does not conflict with the result that BMP3 is positively selected (Canalis et al., 2003). Therefore, similar to the role of BMP3 in the development of intermuscular bone in blunt snout bream (Zhang et al., 2018), we speculate that BMP3 may act as a negative regulator of skeletal growth, similarly to other positive BMPs, during the formation and development of the beak-like teeth and maintain internal stability. Evolutionary selection pressure analysis revealed a positively selected site in BMP7 under the branch-site model. Positive selection pressure on these loci may be an important reason for the tissue-specific expression and functional diversity of BMP genes. Studies have shown that BMP7 is an important initiation signal molecule for tooth germ development. It is secreted by epithelial cells and induces the expression of mesenchyme. In addition, BMP7 can participate in the formation of tooth germ and mediate the differentiation of mesenchymal cells (Tasli et al., 2014; Gao et al., 2015). Furthermore, studies on mouse teeth suggest that BMP2, BMP4, and BMP7 play a role in regulating tooth eruption and shape development and may be involved in the induction and formation of dentin and enamel (Aberg et al., 1997). Therefore, it is inferred that BMP7 plays an important role in the development of the special beak-like teeth of *O. fasciatus* and *O. punctatus*, which distinguish them from other species evolutionarily.

## Conclusion

In summary, we identified a total of 19 BMP genes in the *O. fasciatus* genome, and 16 members of the BMP gene family were identified in *O. punctatus,* with BMP1, BMP12 and BMP14 being lost. Subsequently, to gain a comprehensive and deep understanding of the BMP gene family and its distribution in the genome, we performed phylogenetic analysis, BMP sequence alignment, motif and domain composition analysis, collinearity analysis, and selection pressure analysis, among other analyses. We also described the expression patterns of BMPs in the gills, liver, spleen, brain, the surrounding tissues of beak-like teeth teeth, intestine, muscle, and heart of *O. punctatus*. Most of the BMP genes were widely expressed. The results on the temporal regulation of these BMP genes during tooth development in *O. punctatus* are notable. The expression of BMPs significantly increased from 16 to 30 dph, which indicates that osteoblasts are rapidly generated at 30 dph and that activation of osteoblasts in *O. punctatus* occurs in preparation for ossification of the bones and beak-like teeth at 40–50 dph. Our investigation revealed the effects of the BMP gene family on the healing and ossification development of the beak-like teeth of *O. fasciatus* and *O. punctatus* at the levels of genome evolution and RNA molecules, providing an immeasurable reference for future research on the molecular biology, physiology, and evolution of *Oplegnathus*. However, the detailed functions and regulatory mechanisms of each BMP gene still require further exploration.

## Data Availability

The original contributions presented in the study are included in the article/Supplementary Material, further inquiries can be directed to the corresponding authors.
