# Supplementary material for: Genome-wide identification, characterization and expression analysis of the BMP family associated with beak-like teeth in Oplegnathus
Source: Front Genet. 2022 Jul 18;13:938473. doi: 10.3389/fgene.2022.938473 (PMC9342863; doi:10.3389/fgene.2022.938473)
Supplement: Supplementary file 1 [file DataSheet1.ZIP › Table S1. Primers used for expression profiling.docx]

Table S1 Primers used for expression profiling.

| Gene | F5’-3’ | R5’-3’ | size(bp) |
| --- | --- | --- | --- |
| β-actin  OpBMP2 | GCTGTGCTGTCCCTGTA  GTAGTAGGCACGTCAGGGTG | GAGTAGCCACGCTCTGTC  CGTTTTTCCCGTGTGTGGAG | 130 |
| OpBMP3b-1 | GGTGCTGAGCTTCGATGAGA | AAGGACGCACCACTTTAGGG | 200 |
| OpBMP3b-2 | ATGAGCTGCCTGAGGTTGAC | AGGTTTGAGCCCCTGTGATG | 209 |
| OpBMP4 | GACGGATTGGAAACCTCGCT | TTGGGAACAGGCAATGGACA | 137 |
| OpBMP6 | CCAGGCCCAGCAGATTACAA | AATGATCCAGTCCTGCCAGC | 102 |
| OpBMP7a | TGGAGGCGGCAACAGATAAC | ATAGCGTGGTTGGTGGCATT | 187 |
| OpBMP7b | ATCAATCCTCGTGTGGCAGG | GGAGTGCCTCTTGGCTCTTT | 178 |
| OpBMP8 | GAGTCCTGCCAACAGTGGAG | GAATATCCAGTGGGGGCCAA | 105 |
| OpBMP9 | GGAGATGGGCTTCCAAACGA | GTAACACAGGCCTCGACACT | 180 |
| OpBMP10 | CTGCGAGAGGCGATGGATTTAGAG | AGCGTTGAACAGCAGCAGTGAG | 126 |
| OpBMP11-1 | TTCAAACAGCCCCACACCAA | ACAGTCAGAGGGTAGCGACA | 215 |
| OpBMP11-2 | CCAACTGGGGCATCGAGATT | GCGCTTTGGGGCAATAATCC | 248 |
| OpBMP11-3 | GTGAAGATCCTCGACAGCCC | CTGCAGGTGCATAAACTCGC | 195 |
| OpBMP16 | AGAGCCGAGGGATCGTAACA | CAGACGAGGCTTGAGGTAGC | 138 |
