# Supplementary material for: Genome-wide identification, characterization and expression analysis of the BMP family associated with beak-like teeth in Oplegnathus
Source: Front Genet. 2022 Jul 18;13:938473. doi: 10.3389/fgene.2022.938473 (PMC9342863; doi:10.3389/fgene.2022.938473)
Supplement: Supplementary file 1 [file DataSheet1.ZIP › Table S10. BMP8 model parameter estimates and log-likelihoods.docx]

Table S10. BMP8 model parameter estimates and log-likelihoods

|  | Model | np | lnL | omega | Positive selection  site(BEB) |
| --- | --- | --- | --- | --- | --- |
| Branch model | one ratio | 23 | -18743.366166 | 0.5161 | None |
|  | two ratio | 24 | -18742.981908 | 0.52416 0.00211 | None |
|  | free ratio | 43 | -18720.834591 | 344.20507 73.36234 0.08798 0.20544 440.39469 0.06226 392.27044 0.00010 1.12972 1.05199 2.59889 1.34525 30.08189 2.16934 578.37930 7.06896 0.00010 0.26775 0.01591 2.14254 0.47852 | None |
| Site model | M0 | 23 | -18743.366166 | 0.5161 | None |
|  | M1a | 24 | -18727.169820 | p: 0.00225 0.99775  w: 0.00000 1.00000 | None |
|  | M2a | 26 | -18727.158310 | p: 0.00225 0.90920 0.08854  w: 0.00000 1.00000 1.00000 | None |
|  | M3 | 27 | -18716.976315 | p: 0.00070 0.00156 0.99774  w: 0.00000 0.00000 0.51309 | None |
|  | M7 | 24 | -18725.118768 | p =1.33398 q =1.43949 | None |
|  | M8 | 26 | -18727.158292 | p0 =0.00225 p =0.00500 q =2.58262  (p1 =0.99775) w =1.00000 | None |
| Branch-site model | M0 | 25 | -18727.158299 | site class 0 1 2a 2b  proportion 0.00225 0.99651 0.00000 0.00124  background w 0.00000 1.00000 0.00000 1.00000  foreground w 0.00000 1.00000 1.00000 1.00000 | None |
|  | MA | 26 | -18727.157416 | site class 0 1 2a 2b  proportion 0.00225 0.99775 0.00000 0.00000  background w 0.00000 1.00000 0.00000 1.00000  foreground w 0.00000 1.00000 1.00000 1.00000 | None |
