# Supplementary material for: Genome-wide identification, characterization and expression analysis of the BMP family associated with beak-like teeth in Oplegnathus
Source: Front Genet. 2022 Jul 18;13:938473. doi: 10.3389/fgene.2022.938473 (PMC9342863; doi:10.3389/fgene.2022.938473)
Supplement: Supplementary file 1 [file DataSheet1.ZIP › Table S11. BMP10 model parameter estimates and log-likelihoods.docx]

Table S11. BMP10 model parameter estimates and log-likelihoods

|  | Model | np | lnL | omega | Positive selection  site(BEB) |
| --- | --- | --- | --- | --- | --- |
| Branch model | one ratio | 27 | -13165.315119 | 0.14579 | None |
|  | two ratio-10 | 28 | -13165.044492 | 0.14377 0.18619 | None |
|  | two ratio-10a | 28 | -13165.305936 | 0.14559 0.15295 | None |
|  | free ratio | 51 | -13124.407179 | 0.00649 0.00102 0.11175 0.13685 0.19728 0.24741 0.30299 0.15699 0.16859 0.15976 0.41728 0.11127 0.27309 0.32206 112.97213 0.13366 0.02903 0.07065 0.08406 0.07704 0.22694 0.14512 0.21308 0.32356 2.18933 | None |
| Site model | M0 | 27 | -13165.315119 | 0.14579 | None |
|  | M1a | 28 | -12906.854721 | p: 0.69044 0.30956  w: 0.08859 1.00000 | None |
|  | M2a | 30 | -12906.854721 | p: 0.69044 0.09885 0.21071  w: 0.08859 1.00000 1.00000 | None |
|  | M3 | 31 | -12787.169522 | p: 0.27688 0.39723 0.32589  w: 0.01295 0.10143 0.45828 | None |
|  | M7 | 28 | -12784.975567 | p =0.55456 q =2.24189 | None |
|  | M8 | 30 | -12775.021631 | p0 =0.99312 p =0.57971 q =2.48464  (p1 =0.00688) w =24.72441 | None |
| Branch-site model | M0-10 | 29 | -12906.854721 | site class 0 1 2a 2b  proportion 0.69044 0.30956 0.00000 0.00000  background w 0.08859 1.00000 0.08859 1.00000  foreground w 0.08859 1.00000 1.00000 1.00000 | None |
|  | MA-10 | 30 | -12906.854721 | site class 0 1 2a 2b  proportion 0.69044 0.30956 0.00000 0.00000  background w 0.08859 1.00000 0.08859 1.00000  foreground w 0.08859 1.00000 1.00000 1.00000 | None |
|  | M0-10a | 29 | -12896.862800 | site class 0 1 2a 2b  proportion 0.50701 0.22421 0.18637 0.08241  background w 0.08280 1.00000 0.08280 1.00000  foreground w 0.08280 1.00000 1.00000 1.00000 | None |
|  | MA-10a | 30 | -12896.804865 | site class 0 1 2a 2b  proportion 0.52319 0.23087 0.17063 0.07530  background w 0.08294 1.00000 0.08294 1.00000  foreground w 0.08294 1.00000 1.26086 1.26086 | None |
