# Supplementary material for: Genome-wide identification, characterization and expression analysis of the BMP family associated with beak-like teeth in Oplegnathus
Source: Front Genet. 2022 Jul 18;13:938473. doi: 10.3389/fgene.2022.938473 (PMC9342863; doi:10.3389/fgene.2022.938473)
Supplement: Supplementary file 1 [file DataSheet1.ZIP › Table S12. BMP11 model parameter estimates and log-likelihoods.docx]

Table S12. BMP11 model parameter estimates and log-likelihoods

|  | Model | np | lnL | omega | Positive selection  site(BEB) |
| --- | --- | --- | --- | --- | --- |
| Branch model | one ratio | 29 | -7906.271958 | 0.02097 | None |
|  | two ratio-1 | 30 | -7903.814685 | 0.02221 0.00010 | None |
|  | two ratio-23 | 30 | -7903.675956 | 0.02189 999.00000 | None |
|  | free ratio | 55 | -7865.867236 | 0.53895 0.00621 0.05476 0.00010 0.07688 0.00010 0.01874 0.02336 0.01450 0.06281 0.08470 0.09597 0.11081 30.72917 0.03801 0.00619 0.00981 0.11321 0.00479 0.01149 0.97100 0.04923 0.00010 0.00010 0.00302 0.00010 0.02862 | None |
| Site model | M0 | 29 | -7906.271958 | 0.02097 | None |
|  | M1a | 30 | -7817.049622 | p: 0.91779 0.08221  w: 0.01058 1.00000 | None |
|  | M2a | 32 | -7817.049622 | p: 0.91779 0.04215 0.04006  w: 0.01058 1.00000 1.00000 | None |
|  | M3 | 33 | -7664.514036 | p: 0.55889 0.32979 0.11132  w: 0.00151 0.02210 0.16134 | None |
|  | M7 | 30 | -7669.777386 | p =0.28783 q =9.73926 | None |
|  | M8 | 32 | -7669.781376 | p0 =0.99999 p =0.28783 q =9.73926  (p1 =0.00001) w =44.26493 | None |
| Branch-site model | M0-1 | 31 | -7817.049623 | site class 0 1 2a 2b  proportion 0.91779 0.08221 0.00000 0.00000  background w 0.01058 1.00000 0.01058 1.00000  foreground w 0.01058 1.00000 1.00000 1.00000 | None |
|  | MA-1 | 32 | -7817.049621 | site class 0 1 2a 2b  proportion 0.91779 0.08221 0.00000 0.00000  background w 0.01057 1.00000 0.01057 1.00000  foreground w 0.01057 1.00000 1.00000 1.00000 | None |
|  | M0-23 | 31 | -7807.510700 | site class 0 1 2a 2b  proportion 0.64605 0.05829 0.27120 0.02447  background w 0.01154 1.00000 0.01154 1.00000  foreground w 0.01154 1.00000 1.00000 1.00000 | None |
|  | MA-23 | 32 | -7807.086185 | site class 0 1 2a 2b  proportion 0.62757 0.05669 0.28958 0.02616  background w 0.01189 1.00000 0.01189 1.00000  foreground w 0.01189 1.00000 999.00000 999.00000 | None |
