# Supplementary material for: Genome-wide identification, characterization and expression analysis of the BMP family associated with beak-like teeth in Oplegnathus
Source: Front Genet. 2022 Jul 18;13:938473. doi: 10.3389/fgene.2022.938473 (PMC9342863; doi:10.3389/fgene.2022.938473)
Supplement: Supplementary file 1 [file DataSheet1.ZIP › Table S13. BMP13 model parameter estimates and log-likelihoods.docx]

Table S13. BMP13 model parameter estimates and log-likelihoods

|  | Model | np | lnL | omega | Positive selection  site(BEB) |
| --- | --- | --- | --- | --- | --- |
| Branch model | one ratio | 27 | -10821.782623 | 0.12511 | None |
|  | two ratio-Of | 28 | -10820.505655 | 0.12834 0.07933 | None |
|  | two ratio-Op | 28 | -10819.51681 | 0.12951 0.06348 | None |
|  | free ratio | 51 | -10781.453679 | 146.32728 0.19047 0.06642 0.14039 0.22301 0.10186 0.10292 0.06756 0.17632 0.12554 0.17725 0.16252 0.08248 0.26526 0.14174 0.09487 0.08446 0.18304 0.09819 0.69852 0.00261 0.09606 999.00000 0.07594 0.20850 | None |
| Site model | M0 | 27 | -10821.782623 | 0.12511 | None |
|  | M1a | 28 | -10620.343273 | p: 0.72008 0.27992  w: 0.07266 1.00000 | None |
|  | M2a | 30 | -10620.343331 | p: 0.72008 0.27992 0.00000  w: 0.07266 1.00000 102.38708 | None |
|  | M3 | 31 | -10478.420555 | p: 0.40657 0.31383 0.27960  w: 0.00893 0.12801 0.44141 | None |
|  | M7 | 28 | -10479.680799 | p =0.39978 q =1.90140 | None |
|  | M8 | 30 | -10479.631483 | p0 =0.99842 p =0.40263 q =1.93811  (p1 =0.00158) w =1.96237 | None |
| Branch-site model | M0-Of | 29 | -10620.343273 | site class 0 1 2a 2b  proportion 0.72008 0.27992 0.00000 0.00000  background w 0.07266 1.00000 0.07266 1.00000  foreground w 0.07266 1.00000 1.00000 1.00000 | None |
|  | MA-Of | 30 | -10620.17311 | site class 0 1 2a 2b  proportion 0.71702 0.27950 0.00250 0.00098  background w 0.07223 1.00000 0.07223 1.00000  foreground w 0.07223 1.00000 4.29902 4.29902 | None |
|  | M0-Op | 29 | -10619.728544 | site class 0 1 2a 2b  proportion 0.70839 0.27606 0.01119 0.00436  background w 0.07183 1.00000 0.07183 1.00000  foreground w 0.07183 1.00000 1.00000 1.00000 | None |
|  | MA-Op | 30 | -10620.343272 | site class 0 1 2a 2b  proportion 0.72008 0.27992 0.00000 0.00000  background w 0.07266 1.00000 0.07266 1.00000  foreground w 0.07266 1.00000 18.49938 18.49938 | None |
