# Supplementary material for: Genome-wide identification, characterization and expression analysis of the BMP family associated with beak-like teeth in Oplegnathus
Source: Front Genet. 2022 Jul 18;13:938473. doi: 10.3389/fgene.2022.938473 (PMC9342863; doi:10.3389/fgene.2022.938473)
Supplement: Supplementary file 1 [file DataSheet1.ZIP › Table S14. BMP15 model parameter estimates and log-likelihoods.docx]

Table S14. BMP15 model parameter estimates and log-likelihoods

|  | Model | np | lnL | omega | Positive selection  site(BEB) |
| --- | --- | --- | --- | --- | --- |
| Branch model | one ratio | 23 | -12088.469985 | 0.19855 | None |
|  | two ratio | 24 | -12085.794169 | 0.21011 0.07200 | None |
|  | free ratio | 43 | -12032.152671 | 999.00000 0.04968 0.06540 0.13337 0.08763 0.05242 0.25345 0.28569 0.07668 0.00185 0.30040 0.21966 0.53869 0.21084 999.00000 0.00338 999.00000 0.13870 0.11211 0.55992 0.31532 | None |
| Site model | M0 | 23 | -12088.469985 | 0.19855 | None |
|  | M1a | 24 | -11930.443325 | p: 0.62675 0.37325  w: 0.13672 1.00000 | None |
|  | M2a | 26 | -11930.443325 | p: 0.62675 0.00000 0.37325  w: 0.13672 1.00000 1.00000 | None |
|  | M3 | 27 | -11837.918893 | p: 0.20651 0.43022 0.36327  w: 0.02075 0.15737 0.52284 | None |
|  | M7 | 24 | -11836.628087 | p =0.78788 q =2.15614 | None |
|  | M8 | 26 | -11834.874316 | p0 =0.99263 p =0.80567 q =2.27588  (p1 =0.00737) w =4.37467 | None |
| Branch-site model | M0 | 25 | -11930.443325 | site class 0 1 2a 2b  proportion 0.62675 0.37325 0.00000 0.00000  background w 0.13672 1.00000 0.13672 1.00000  foreground w 0.13672 1.00000 1.00000 1.00000 | None |
|  | MA | 26 | -11930.443325 | site class 0 1 2a 2b  proportion 0.62675 0.37325 0.00000 0.00000  background w 0.13672 1.00000 0.13672 1.00000  foreground w 0.13672 1.00000 1.00000 1.00000 | None |
