# Supplementary material for: Genome-wide identification, characterization and expression analysis of the BMP family associated with beak-like teeth in Oplegnathus
Source: Front Genet. 2022 Jul 18;13:938473. doi: 10.3389/fgene.2022.938473 (PMC9342863; doi:10.3389/fgene.2022.938473)
Supplement: Supplementary file 1 [file DataSheet1.ZIP › Table S15. BMP16 model parameter estimates and log-likelihoods.docx]

Table S15. BMP16 model parameter estimates and log-likelihoods

|  | Model | np | lnL | omega | Positive selection  site(BEB) |
| --- | --- | --- | --- | --- | --- |
| Branch model | one ratio | 13 | -6307.525379 | 0.18513 | None |
|  | two ratio | 14 | -6306.753551 | 0.17645 0.39083 | None |
|  | free ratio | 23 | -6293.045638 | 0.21396 0.21842 2.83592 0.00010 0.24299 0.21281 0.06798 0.12855 999.00000 0.08515 0.40252 | None |
| Site model | M0 | 13 | -6307.525379 | 0.18513 | None |
|  | M1a | 14 | -6212.626202 | p: 0.71273 0.28727  w: 0.07829 1.00000 | None |
|  | M2a | 16 | -6212.626202 | p: 0.71273 0.18269 0.10458  w: 0.07829 1.00000 1.00000 | None |
|  | M3 | 17 | -6203.602252 | p: 0.61470 0.34709 0.03822  w: 0.04843 0.52032 1.54214 | None |
|  | M7 | 14 | -6206.927569 | p =0.37772 q =1.11534 | None |
|  | M8 | 16 | -6205.757096 | p0 =0.87315 p =0.51812 q =2.59524  (p1 =0.12685) w =1.00000 | None |
| Branch-site model | M0 | 15 | -6212.626202 | site class 0 1 2a 2b  proportion 0.71273 0.28727 0.00000 0.00000  background w 0.07829 1.00000 0.07829 1.00000  foreground w 0.07829 1.00000 1.00000 1.00000 | None |
|  | MA | 16 | -6212.625579 | site class 0 1 2a 2b  proportion 0.71260 0.28722 0.00013 0.00005  background w 0.07829 1.00000 0.07829 1.00000  foreground w 0.07829 1.00000 1.08479 1.08479 | None |
