# Supplementary material for: Genome-wide identification, characterization and expression analysis of the BMP family associated with beak-like teeth in Oplegnathus
Source: Front Genet. 2022 Jul 18;13:938473. doi: 10.3389/fgene.2022.938473 (PMC9342863; doi:10.3389/fgene.2022.938473)
Supplement: Supplementary file 1 [file DataSheet1.ZIP › Table S16. Likelihood ratio test statistic for BMP.docx]

Table S16. Likelihood ratio test statistic for BMP

|  | Model | Model comparison | df | LRT（2ΔL） | P | Accepted model |
| --- | --- | --- | --- | --- | --- | --- |
| BMP2 | Branch model | M0 vs M1 | 28 | 479.092468 | 0.00000000 | M1 |
|  |  | M0 vs M2 | 1 | 2.788658 | 0.09493714 | M0 |
|  | Site model | M0 vs M3 | 4 | 504.49659 | 0.00000000 | M3 |
|  |  | M1a vs M2a | 2 | 0 | 1.00000000 | M1a |
|  |  | M7 vs M8 | 0 | 0.679624 | 0.40972450 | M7 |
|  | Branch-site model | MA vs null | 1 | 1.489714 | 0.22226301 | null |
| BMP4 | Branch model | M0 vs M1 | 18 | 33.909306 | 0.01292427 | M1 |
|  |  | M0 vs M2 | 1 | 2.392204 | 0.12194191 | M0 |
|  | Site model | M0 vs M3 | 4 | 385.709656 | 0.00000000 | M3 |
|  |  | M1a vs M2a | 1 | 4.00E-06 | 1.00000000 | M1a |
|  |  | M7 vs M8 | 2 | 0.000488 | 0.99980002 | M7 |
|  | Branch-site model | MA vs null | 1 | 0.00002 | 1.00000000 | null |
| BMP5 | Branch model | M0 vs M1 | 18 | 23.721966 | 0.164351544 | M0 |
|  |  | M0 vs M2 | 1 | 1.418278 | 0.233699902 | M0 |
|  | Site model | M0 vs M3 | 4 | 530.56681 | 0.000000000 | M3 |
|  |  | M1a vs M2a | 2 | 2.60E-05 | 1.000000000 | M2a |
|  |  | M7 vs M8 | 2 | 0.494468 | 0.780984481 | M7 |
|  | Branch-site model | MA vs null | 1 | 0 | 1.000000000 | null |
| BMP6 | Branch model | M0 vs M1 | 20 | 94.070728 | 0.00000000 | M1 |
|  |  | M0 vs M2 | 1 | 1.156072 | 0.28227584 | M0 |
|  | Site model | M0 vs M3 | 4 | 495.809678 | 0.00000000 | M3 |
|  |  | M1a vs M2a | 2 | 0 | 1.00000000 | M2a |
|  |  | M7 vs M8 | 2 | 6.463408 | 0.03949031 | M8 |
|  | Branch-site model | MA vs null | 1 | 4.166894 | 0.041223589 | MA |
| BMP8 | Branch model | M0 vs M1 | 20 | 45.06315 | 0.00108193 | M1 |
|  |  | M0 vs M2 | 1 | 0.768516 | 0.38068149 | M0 |
|  | Site model | M0 vs M3 | 4 | 32.415712 | 0.00000157 | M3 |
|  |  | M1a vs M2a | 2 | 2.30E-02 | 0.98856587 | M1a |
|  |  | M7 vs M8 | 2 | 4.079048 | 0.13009374 | M7 |
|  | Branch-site model | MA vs null | 1 | 0.001766 | 0.96711170 | null |
| BMP10 | Branch model | M0 vs M1 | 24 | 81.81588 | 0.00000003 | M1 |
|  |  | M0 vs M2-10 | 1 | 0.541254 | 0.46193583 | M0 |
|  |  | M0 vs M2-10a | 1 | 0.018366 | 0.89239248 | M0 |
|  | Site model | M0 vs M3 | 4 | 756.291194 | 0.00000000 | M3 |
|  |  | M1a vs M2a | 2 | 0 | 1.00000000 | M1a |
|  |  | M7 vs M8 | 2 | 19.907872 | 0.00004754 | M8 |
|  | Branch-site model | MA vs null-10 | 1 | 0 | 1.00000000 | null |
|  |  | MA vs null-10a | 1 | 0.11587 | 0.73363514 | null |
| BMP11 | Branch model | M0 vs M1 | 26 | 80.809444 | 0.00000016 | M1 |
|  |  | M0 vs M2-1 | 1 | 4.914546 | 0.02663217 | M2 |
|  |  | M0 vs M2-23 | 1 | 5.192004 | 0.02269109 | M2 |
|  | Site model | M0 vs M3 | 4 | 483.515844 | 0.00000000 | M3 |
|  |  | M1a vs M2a | 2 | 0 | 1.00000000 | M1a |
|  |  | M7 vs M8 | 2 | 0.00798 | 0.99605779 | M7 |
|  | Branch-site model | MA vs null-1 | 1 | 0.000004 | 1.00000000 | null |
|  |  | MA vs null-23 | 1 | 0.84903 | 0.35683539 | null |
| BMP13 | Branch model | M0 vs M1 | 24 | 80.657888 | 0.00000005 | M1 |
|  |  | M0 vs M2-Of | 1 | 2.553936 | 0.11002237 | M0 |
|  |  | M0 vs M2-Op | 1 | 4.531634 | 0.03327449 | M2 |
|  | Site model | M0 vs M3 | 4 | 686.724136 | 0.00000000 | M3 |
|  |  | M1a vs M2a | 2 | 1.16E-04 | 0.99995000 | M1a |
|  |  | M7 vs M8 | 2 | 0.098632 | 0.95189552 | M7 |
|  | Branch-site model | MA vs null-Of | 1 | 0.340322 | 0.55965611 | null |
|  |  | MA vs null-Op | 1 | 1.229456 | 0.26752376 | null |
| BMP15 | Branch model | M0 vs M1 | 20 | 112.634628 | 0.00000000 | M1 |
|  |  | M0 vs M2 | 1 | 5.351632 | 0.02070326 | M2 |
|  | Site model | M0 vs M3 | 4 | 501.102184 | 0.00000000 | M3 |
|  |  | M1a vs M2a | 2 | 0 | 1.00000000 | M1a |
|  |  | M7 vs M8 | 2 | 3.507542 | 0.17312351 | M7 |
|  | Branch-site model | MA vs null | 1 | 0 | 1.00000000 | null |
| BMP16 | Branch model | M0 vs M1 | 10 | 28.959482 | 0.00126504 | M1 |
|  |  | M0 vs M2 | 1 | 1.543656 | 0.21408275 | M0 |
|  | Site model | M0 vs M3 | 4 | 207.846254 | 0.00000000 | M3 |
|  |  | M1a vs M2a | 2 | 0 | 1.00000000 | M1a |
|  |  | M7 vs M8 | 2 | 2.340946 | 0.31022731 | M7 |
|  | Branch-site model | MA vs null | 1 | 0.001246 | 0.97236600 | null |
