# Supplementary material for: Genome-wide identification, characterization and expression analysis of the BMP family associated with beak-like teeth in Oplegnathus
Source: Front Genet. 2022 Jul 18;13:938473. doi: 10.3389/fgene.2022.938473 (PMC9342863; doi:10.3389/fgene.2022.938473)
Supplement: Supplementary file 1 [file DataSheet1.ZIP › Table S2. The species and corresponding accession numbers..docx]

Table S2. The species and corresponding accession numbers.

| Species | Gene | Accession number |
| --- | --- | --- |
| Cyprinus carpio | BMP1a-1 | LC219865 |
| Cyprinus carpio | BMP1a-2 | LC219866 |
| Cyprinus carpio | BMP1b-1 | LC219867 |
| Cyprinus carpio | BMP1b-2 | LC219868 |
| Cyprinus carpio | BMP2a-1 | LC219869 |
| Cyprinus carpio | BMP2a-2 | LC219870 |
| Cyprinus carpio | BMP2b-1 | LC219871 |
| Cyprinus carpio | BMP2b-2 | LC219872 |
| Cyprinus carpio | BMP3a-1 | LC219873 |
| Cyprinus carpio | BMP3a-2 | LC219874 |
| Cyprinus carpio | BMP3b-1 | LC219875 |
| Cyprinus carpio | BMP3b-2 | LC219876 |
| Cyprinus carpio | BMP4-1 | LC219877 |
| Cyprinus carpio | BMP4-2 | LC219878 |
| Cyprinus carpio | BMP5-1 | LC219879 |
| Cyprinus carpio | BMP5-2 | LC219880 |
| Cyprinus carpio | BMP6a-1 | LC219881 |
| Cyprinus carpio | BMP6a-2 | LC219882 |
| Cyprinus carpio | BMP7a-1 | LC219883 |
| Cyprinus carpio | BMP7a-2 | LC219884 |
| Cyprinus carpio | BMP7b-1 | LC219885 |
| Cyprinus carpio | BMP7b-2 | LC219886 |
| Cyprinus carpio | BMP8a-1 | LC219887 |
| Cyprinus carpio | BMP8a-2 | LC221568 |
| Cyprinus carpio | BMP9-1 | LC219888 |
| Cyprinus carpio | BMP9-2 | LC219889 |
| Cyprinus carpio | BMP10a-1 | LC221569 |
| Cyprinus carpio | BMP10a-2 | LC221570 |
| Cyprinus carpio | BMP10b-1 | LC219890 |
| Cyprinus carpio | BMP10b-2 | LC219891 |
| Cyprinus carpio | BMP11-1 | LC219892 |
| Cyprinus carpio | BMP11-2 | LC219893 |
| Cyprinus carpio | BMP12-1 | LC219894 |
| Cyprinus carpio | BMP12-2 | LC219895 |
| Cyprinus carpio | BMP13a-1 | LC219896 |
| Cyprinus carpio | BMP13a-2 | LC219897 |
| Cyprinus carpio | BMP13b-1 | LC219898 |
| Cyprinus carpio | BMP13b-2 | LC219899 |
| Cyprinus carpio | BMP14-1 | LC219900 |
| Cyprinus carpio | BMP14-2 | LC219901 |
| Cyprinus carpio | BMP15-1 | LC219861 |
| Cyprinus carpio | BMP15-2 | LC219862 |
| Cyprinus carpio | BMP16-1 | LC219863 |
| Cyprinus carpio | BMP16-2 | LC219864 |
| Salmo salar | BMP2 | NP_001167305.1 |
| Salmo salar | BMP3 | XM_014172740.1 |
| Salmo salar | BMP4 | NP_001133316.1 |
| Salmo salar | BMP5 | ENSSSAG00000095517 |
| Salmo salar | BMP6 | XM_014181053.1 |
| Salmo salar | BMP12 | XM_014211035.1 |
| Salmo salar | BMP13 | XM_014177002.1 |
| Salmo salar | BMP15 | XP_014012180.1 |
| Salmo salar | BMP16 | XP_014068936.1 |
| Danio rerio | BMP1a | ENSDARG00000028071 |
| Danio rerio | BMP2a | ENSDARG00000013409 |
| Danio rerio | BMP2b | ENSDARG00000041430 |
| Danio rerio | BMP3 | ENSDARG00000060526 |
| Danio rerio | BMP3b | ENSDARG00000073891 |
| Danio rerio | BMP4 | ENSDARG00000019995 |
| Danio rerio | BMP5 | ENSDARG00000101701 |
| Danio rerio | BMP6 | ENSDARG00000015686 |
| Danio rerio | BMP7a | ENSDARG00000018260 |
| Danio rerio | BMP7b | ENSDARG00000063230 |
| Danio rerio | BMP8a | ENSDARG00000035677 |
| Danio rerio | BMP9 | ENSDART00000082220 |
| Danio rerio | BMP10 | ENSDARG00000061769 |
| Danio rerio | BMP11 | ENSDARG00000044924 |
| Danio rerio | BMP12 | ENSDARG00000042784 |
| Danio rerio | BMP13a | ENSDARG00000053479 |
| Danio rerio | BMP13b | ENSDARG00000005510 |
| Danio rerio | BMP14 | ENSDART00000015051 |
| Danio rerio | BMP15 | ENSDARG00000037491 |
| Danio rerio | BMP16 | ENSDARG00000103679 |
| Oryzias latipes | BMP1 | ENSORLG00000011156 |
| Oryzias latipes | BMP2 | ENSORLG00000009772 |
| Oryzias latipes | BMP3 | ENSORLG00000017782 |
| Oryzias latipes | BMP4 | ENSORLG00000013304 |
| Oryzias latipes | BMP5 | ENSORLG00000009907 |
| Oryzias latipes | BMP6 | ENSORLG00000006529 |
| Oryzias latipes | BMP7b | ENSORLG00000011720 |
| Oryzias latipes | BMP8a | ENSORLG00000007124 |
| Oryzias latipes | BMP9 | ENSORLG00000020287 |
| Oryzias latipes | BMP10 | ENSORLG00000015531 |
| Oryzias latipes | BMP11 | ENSORLG00000014837 |
| Oryzias latipes | BMP13a | ENSORLG00000020688 |
| Oryzias latipes | BMP13b | ENSORLG00000007867 |
| Oryzias latipes | BMP14 | ENSORLG00000002972 |
| Oryzias latipes | BMP15 | ENSORLG00000008622 |
| Xenopus laevis | BMP1 | ENSXLAG00005018738 |
| Xenopus laevis | BMP2a | ENSXLAG00005027501 |
| Xenopus laevis | BMP2b | ENSXLAG00005010312 |
| Xenopus laevis | BMP3 | ENSXLAG00005015543 |
| Xenopus laevis | BMP4 | ENSXLAG00005003605 |
| Xenopus laevis | BMP5 | ENSXLAG00005027196 |
| Xenopus laevis | BMP6 | ENSXLAG00005004879 |
| Xenopus laevis | BMP7 | ENSXLAG00005001862 |
| Xenopus laevis | BMP8 | ENSXLAG00005002224 |
| Xenopus laevis | BMP9 | ENSXLAG00005026171 |
| Xenopus laevis | BMP10 | ENSXLAG00005001110 |
| Xenopus laevis | BMP11 | ENSXLAG00005006861 |
| Xenopus laevis | BMP12 | ENSXLAG00005018104 |
| Xenopus laevis | BMP15 | ENSXLAG00005027319 |
| Takifugu rubripes | BMP1 | ENSTRUG00000002403 |
| Takifugu rubripes | BMP2b | ENSTRUG00000007962 |
| Takifugu rubripes | BMP3 | ENSTRUG00000000901 |
| Takifugu rubripes | BMP4 | ENSTRUG00000014535 |
| Takifugu rubripes | BMP5 | ENSTRUG00000015401 |
| Takifugu rubripes | BMP6 | ENSTRUG00000006494 |
| Takifugu rubripes | BMP7a | ENSTRUG00000011215 |
| Takifugu rubripes | BMP7b | ENSTRUG00000003888 |
| Takifugu rubripes | BMP8a | ENSTRUG00000005036 |
| Takifugu rubripes | BMP9 | ENSTRUG00000017118 |
| Takifugu rubripes | BMP10 | ENSTRUG00000015051 |
| Takifugu rubripes | BMP11 | ENSTRUG00000018458 |
| Takifugu rubripes | BMP13 | ENSTRUG00000004399 |
| Takifugu rubripes | BMP15 | ENSTRUG00000007065 |
| Takifugu rubripes | BMP16 | ENSTRUG00000011486 |
| Gallus gallus | BMP1 | AAC02259.1 |
| Gallus gallus | BMP2 | ENSGALG00000029301 |
| Gallus gallus | BMP3 | ENSGALG00000035052 |
| Gallus gallus | BMP3b | ENSGALG00000005985 |
| Gallus gallus | BMP4 | ENSGALG00000012429 |
| Gallus gallus | BMP5 | ENSGALG00000016293 |
| Gallus gallus | BMP6 | XM_015275997.1 |
| Gallus gallus | BMP7 | ENSGALG00000007668 |
| Gallus gallus | BMP9 | ENSGALG00000005981 |
| Gallus gallus | BMP10 | ENSGALG00000000120 |
| Gallus gallus | BMP11 | ENSGALG00000036806 |
| Gallus gallus | BMP13 | ENSGALG00000035901 |
| Gallus gallus | BMP14 | ENSGALG00000038355 |
| Gallus gallus | BMP15 | ENSGALG00000004742 |
| Mus musculus | BMP1 | ENSMUSG00000022098 |
| Mus musculus | BMP2 | ENSMUSG00000027358 |
| Mus musculus | BMP3 | ENSMUSG00000029335 |
| Mus musculus | BMP3b | ENSMUSG00000021943 |
| Mus musculus | BMP4 | ENSMUSG00000021835 |
| Mus musculus | BMP5 | ENSMUSG00000032179 |
| Mus musculus | BMP6 | ENSMUSG00000039004 |
| Mus musculus | BMP7 | ENSMUSG00000008999 |
| Mus musculus | BMP8a | ENSMUSG00000032726 |
| Mus musculus | BMP8b | ENSMUSG00000002384 |
| Mus musculus | BMP9 | ENSMUSG00000072625 |
| Mus musculus | BMP10 | ENSMUSG00000030046 |
| Mus musculus | BMP11 | ENSMUSG00000025352 |
| Mus musculus | BMP12 | ENSMUSG00000037660 |
| Mus musculus | BMP13 | ENSMUSG00000051279 |
| Mus musculus | BMP14 | ENSMUSG00000038259 |
| Mus musculus | BMP15 | ENSMUSG00000023279 |
| Homo sapiens | BMP1 | ENSG00000168487 |
| Homo sapiens | BMP2 | ENSG00000125845 |
| Homo sapiens | BMP3 | ENSG00000152785 |
| Homo sapiens | BMP3b | ENSG00000266524 |
| Homo sapiens | BMP4 | ENSG00000125378 |
| Homo sapiens | BMP5 | ENSG00000112175 |
| Homo sapiens | BMP6 | ENSG00000153162 |
| Homo sapiens | BMP7 | ENSG00000101144 |
| Homo sapiens | BMP8a | ENSG00000183682 |
| Homo sapiens | BMP8b | ENSG00000116985 |
| Homo sapiens | BMP9 | ENSG00000263761 |
| Homo sapiens | BMP10 | ENSG00000163217 |
| Homo sapiens | BMP11 | ENSG00000135414 |
| Homo sapiens | BMP12 | ENSG00000143869 |
| Homo sapiens | BMP13 | ENSG00000156466 |
| Homo sapiens | BMP14 | ENSG00000125965 |
| Homo sapiens | BMP15 | ENSG00000130385 |
