# Supplementary material for: Genome-wide identification, characterization and expression analysis of the BMP family associated with beak-like teeth in Oplegnathus
Source: Front Genet. 2022 Jul 18;13:938473. doi: 10.3389/fgene.2022.938473 (PMC9342863; doi:10.3389/fgene.2022.938473)
Supplement: Supplementary file 1 [file DataSheet1.ZIP › Table S3. BMP3 model parameter estimates and log-likelihoods.docx]

Table S3. BMP3 model parameter estimates and log-likelihoods

|  | Model | np | lnL | omega | Positive selection  site(BEB) |
| --- | --- | --- | --- | --- | --- |
| Branch model | one ratio | 39 | -19580.613334 | 0.11703 | None |
|  | two ratio-3a | 40 | -19577.340768 | 0.11324 0.24294 | None |
|  | two ratio-3b | 40 | -19580.606921 | 0.11696 0.14769 | None |
|  | free ratio | 75 | -19531.620236 | 0.00149 0.00170 0.05641 0.08062 0.10806 0.15559 0.24204 0.08282 0.06634 0.20707 0.10329 0.06957 999.00000 0.10072 0.35015 0.11722 999.00000 0.14461 0.17071 0.00010 0.46060 999.00000 0.00248 999.00000 0.07146 999.00000 0.06307 0.15067 999.00000 999.00000 0.09958 0.00010 0.07790 0.04640 0.15249 0.27753 0.07090 | None |
| Site model | M0 | 39 | -19580.613334 | 0.11703 | None |
|  | M1a | 40 | -19352.013043 | p: 0.70164 0.29836  w: 0.09477 1.00000 | None |
|  | M2a | 42 | -19352.013043 | p: 0.70164 0.19272 0.10563  w: 0.09477 1.00000 1.00000 | None |
|  | M3 | 43 | -19023.651062 | p: 0.35873 0.34748 0.29379  w: 0.02013 0.12985 0.36094 | None |
|  | M7 | 40 | -19036.768000 | p=0.83319 q=4.21692 | None |
|  | M8 | 42 | -19035.464821 | p0=0.98272 p=0.86848 q=4.76436  (p1=0.01728) w=1.00000 | None |
| Branch-site model | M0-3a | 41 | -19337.129624 | site class 0 1 2a 2b  proportion 0.58295 0.25256 0.11477 0.04972  background w 0.09035 1.00000 0.09035 1.00000  foreground w 0.09035 1.00000 1.00000 1.00000 | None |
|  | MA-3a | 42 | -19308.643876 | site class 0 1 2a 2b  proportion 0.66562 0.29522 0.02712 0.01203  background w 0.09040 1.00000 0.09040 1.00000  foreground w 0.09040 1.00000 998.99976 998.99976 | 136 H 0.978*  138 K 0.993**  140 V 0.994**  141 F 0.987*  143 F 0.956*  145 L 0.971*  146 S 0.998**  148 I 0.996**  150 E 0.988*  151 S 0.998**  153 L 0.995** |
|  | M0-3b | 41 | -19349.176374 | site class 0 1 2a 2b  proportion 0.66562 0.29522 0.02712 0.01203  background w 0.09040 1.00000 0.09040 1.00000  foreground w 0.09040 1.00000 998.99976 998.99976 | None |
|  | MA-3b | 42 | -19345.422680 | site class 0 1 2a 2b  proportion 0.61335 0.25146 0.09588 0.03931  background w 0.09463 1.00000 0.09463 1.00000  foreground w 0.09463 1.00000 999.00000 999.00000 | None |
