# Supplementary material for: Genome-wide identification, characterization and expression analysis of the BMP family associated with beak-like teeth in Oplegnathus
Source: Front Genet. 2022 Jul 18;13:938473. doi: 10.3389/fgene.2022.938473 (PMC9342863; doi:10.3389/fgene.2022.938473)
Supplement: Supplementary file 1 [file DataSheet1.ZIP › Table S4. BMP7 model parameter estimates and log-likelihoods.docx]

Table S4. BMP7 model parameter estimates and log-likelihoods

|  | Model | np | lnL | omega | Positive selection  site(BEB) |
| --- | --- | --- | --- | --- | --- |
| Branch model | one ratio | 27 | -10768.548877 | 0.07495 | None |
|  | two ratio-7a | 28 | -10762.934790 | 0.06929 999.00000 | None |
|  | two ratio-7b | 28 | -10767.640160 | 0.07638 0.02807 | None |
|  | free ratio | 57 | -10701.965839 | 39.58984 0.32387 0.04871 0.07363 0.05683 0.00010 0.00010 0.02025 0.06550 0.02958 0.01346 0.04063 0.09964 0.04894 0.03789 118.71015 0.05809 0.67027 0.09873 0.14026 0.18721 0.09552 0.00010 8.58369 0.00429 | None |
| Site model | M0 | 27 | -10768.548877 | 0.07495 | None |
|  | M1a | 28 | -10611.991391 | p: 0.85494 0.14506  w: 0.05643 1.00000 | None |
|  | M2a | 30 | -10611.991391 | p: 0.85494 0.11054 0.03452  w: 0.05643 1.00000 1.00000 | None |
|  | M3 | 31 | -10483.960985 | p: 0.49602 0.37670 0.12729  w: 0.00878 0.11058 0.48073 | None |
|  | M7 | 28 | -10487.103467 | p =0.37847 q =3.09897 | None |
|  | M8 | 30 | -10486.456234 | p0 =0.98273 p =0.40216 q =3.65795  (p1 =0.01727) w =1.00000 | None |
| Branch-site model | M0-7a | 29 | -10611.991391 | site class 0 1 2a 2b  proportion 0.85494 0.14506 0.00000 0.00000  background w 0.05643 1.00000 0.05643 1.00000  foreground w 0.05643 1.00000 1.00000 1.00000 | None |
|  | MA-7a | 30 | -10605.817629 | site class 0 1 2a 2b  proportion 0.66562 0.29522 0.02712 0.01203  proportion 0.00000 0.00000 0.85638 0.14362  background w 0.05220 1.00000 0.05220 1.00000  foreground w 0.05220 1.00000 1.00000 1.00000 | 559 T 0.959* |
|  | M0-7b | 29 | -10611.991391 | site class 0 1 2a 2b  proportion 0.85494 0.14506 0.00000 0.00000  background w 0.05643 1.00000 0.05643 1.00000  foreground w 0.05643 1.00000 1.00000 1.00000 | None |
|  | MA-7b | 30 | -10611.991391 | site class 0 1 2a 2b  proportion 0.85494 0.14506 0.00000 0.00000  background w 0.05643 1.00000 0.05643 1.00000  foreground w 0.05643 1.00000 1.00000 1.00000 | None |
