# Supplementary material for: Genome-wide identification, characterization and expression analysis of the BMP family associated with beak-like teeth in Oplegnathus
Source: Front Genet. 2022 Jul 18;13:938473. doi: 10.3389/fgene.2022.938473 (PMC9342863; doi:10.3389/fgene.2022.938473)
Supplement: Supplementary file 1 [file DataSheet1.ZIP › Table S5. BMP9 model parameter estimates and log-likelihoods.docx]

Table S5. BMP9 model parameter estimates and log-likelihoods

|  | Model | np | lnL | omega | Positive selection  site(BEB) |
| --- | --- | --- | --- | --- | --- |
| Branch model | one ratio | 21 | -11540.222383 | 0.17391 | None |
|  | two ratio | 22 | -11538.120864 | 0.16402 0.51214 | None |
|  | free ratio | 39 | -11506.456581 | 103.56206 0.08814 701.19699 0.35737 0.29730 0.12769 0.12162 0.17205 0.15259 1.01709 0.29993 0.12768 0.15398 999.00000 0.16431 0.06376 0.20157 0.14257 0.00776 | None |
| Site model | M0 | 21 | -11540.222383 | 0.17391 | None |
|  | M1a | 22 | -11270.333721 | p: 0.65141 0.34859  w: 0.09733 1.00000 | None |
|  | M2a | 24 | -11270.333721 | p: 0.65141 0.30963 0.03896  w: 0.09733 1.00000 1.00000 | None |
|  | M3 | 25 | -11191.241450 | p: 0.28304 0.44489 0.27208  w: 0.01979 0.14594 0.71442 | None |
|  | M7 | 22 | -11196.941461 | p =0.57680 q =1.78597 | None |
|  | M8 | 24 | -11187.133908 | p0 =0.85561 p =0.78474 q=4.27385  (p1=0.14439) w=1.03867 | None |
| Branch-site model | M0 | 23 | -11270.333721 | site class 0 1 2a 2b  proportion 0.65141 0.34859 0.00000 0.00000  background w 0.09733 1.00000 0.09733 1.00000  foreground w 0.09733 1.00000 1.00000 1.00000 | None |
|  | MA | 24 | -11259.013051 | site class 0 1 2a 2b  proportion 0.63572 0.33350 0.02018 0.01059  background w 0.09419 1.00000 0.09419 1.00000  foreground w 0.09419 1.00000 317.53626 317.53626 | 26 F 0.996** |
