# Supplementary material for: Genome-wide identification, characterization and expression analysis of the BMP family associated with beak-like teeth in Oplegnathus
Source: Front Genet. 2022 Jul 18;13:938473. doi: 10.3389/fgene.2022.938473 (PMC9342863; doi:10.3389/fgene.2022.938473)
Supplement: Supplementary file 1 [file DataSheet1.ZIP › Table S6. BMP2 model parameter estimates and log-likelihoods.docx]

Table S6. BMP2 model parameter estimates and log-likelihoods

|  | Model | np | lnL | omega | Positive selection  site(BEB) |
| --- | --- | --- | --- | --- | --- |
| Branch model | one ratio | 31 | -7803.676896 | 0.15409 | None |
|  | two ratio | 32 | -7802.282567 | 0.15280 999.00000 | None |
|  | free ratio | 59 | -7564.130662 | 97.79933 0.39634 1.38341 0.56396 0.40064 0.64019 0.45239 0.39197 0.37527 0.43871 0.49262 0.87030 0.42528 0.75173 0.24700 0.52789 0.18106 16.61885 0.60304 0.14026 0.52014 0.20306 0.28689 0.41146 0.39184 1.15242 0.37551 0.00015 0.00010 | None |
| Site model | M0 | 31 | -7803.676896 | 0.15409 | None |
|  | M1a | 32 | -7612.800331 | p: 0.67463 0.32537  w: 0.09290 1.00000 | None |
|  | M2a | 34 | -7612.800331 | p: 0.67463 0.26917 0.05620  w: 0.09290 1.00000 1.00000 | None |
|  | M3 | 35 | -7551.428601 | p: 0.38340 0.40608 0.21053  w: 0.00730 0.18151 0.61926 | None |
|  | M7 | 32 | -7552.713533 | p =0.39730 q =1.48074 | None |
|  | M8 | 32 | -7552.373721 | p0 =0.99576 p =0.40511 q =1.54354  (p1 =0.00424) w =1.80284 | None |
| Branch-site model | M0 | 33 | -7612.623387 | site class 0 1 2a 2b  proportion 0.57696 0.27638 0.09916 0.04750  background w 0.09260 1.00000 0.09260 1.00000  foreground w 0.09260 1.00000 1.00000 1.00000 | None |
|  | MA | 34 | -7611.878530 | site class 0 1 2a 2b  proportio 0.66951 0.32509 0.00363 0.00176  background w 0.09255 1.00000 0.09255 1.00000  foreground w 0.09255 1.00000 999.00000 999.00000 | None |
