# Supplementary material for: Genome-wide identification, characterization and expression analysis of the BMP family associated with beak-like teeth in Oplegnathus
Source: Front Genet. 2022 Jul 18;13:938473. doi: 10.3389/fgene.2022.938473 (PMC9342863; doi:10.3389/fgene.2022.938473)
Supplement: Supplementary file 1 [file DataSheet1.ZIP › Table S7. BMP4 model parameter estimates and log-likelihoods.docx]

Table S7. BMP4 model parameter estimates and log-likelihoods

|  | Model | np | lnL | omega | Positive selection  site(BEB) |
| --- | --- | --- | --- | --- | --- |
| Branch model | one ratio | 21 | -7667.915455 | 0.07322 | None |
|  | two ratio | 22 | -7666.719353 | 0.07177 0.23956 | None |
|  | free ratio | 39 | -7650.960802 | 0.20047 0.06235 0.03645 0.19563 3.41106 0.11939 0.09512 0.10241 0.07105 0.05035 0.13268 0.05029 0.22052 0.06116 0.06425 0.05155 0.05654 0.05458 0.02425 | None |
| Site model | M0 | 21 | -7667.915455 | 0.07322 | None |
|  | M1a | 22 | -7554.897063 | p: 0.87940 0.12060  w: 0.04826 1.00000 | None |
|  | M2a | 24 | -7554.897059 | p: 0.87941 0.10371 0.01688  w: 0.04826 1.00000 1.00000 | None |
|  | M3 | 25 | -7475.060627 | p: 0.48928 0.38047 0.13024  w: 0.00000 0.10234 0.45568 | None |
|  | M7 | 22 | -7477.261580 | p =0.26170 q =2.28068 | None |
|  | M8 | 24 | -7477.261824 | p0 =0.99999 p =0.26169 q =2.28082  (p1 =0.00001) w =1.00003 | None |
| Branch-site model | M0 | 23 | -7554.544590 | site class 0 1 2a 2b  proportion 0.81855 0.11153 0.06153 0.00838  background w 0.04791 1.00000 0.04791 1.00000  foreground w 0.04791 1.00000 1.00000 1.00000 | None |
|  | MA | 24 | -7554.544580 | site class 0 1 2a 2b  proportion 0.81865 0.11154 0.06144 0.00837  background w 0.04791 1.00000 0.04791 1.00000  foreground w 0.04791 1.00000 1.00000 1.00000 | None |
