# Supplementary material for: Genome-wide identification, characterization and expression analysis of the BMP family associated with beak-like teeth in Oplegnathus
Source: Front Genet. 2022 Jul 18;13:938473. doi: 10.3389/fgene.2022.938473 (PMC9342863; doi:10.3389/fgene.2022.938473)
Supplement: Supplementary file 1 [file DataSheet1.ZIP › Table S8. BMP5 model parameter estimates and log-likelihoods.docx]

Table S8. BMP5 model parameter estimates and log-likelihoods

|  | Model | np | lnL | omega | Positive selection  site(BEB) |
| --- | --- | --- | --- | --- | --- |
| Branch model | one ratio | 21 | -8276.691385 | 0.0727 | None |
|  | two ratio | 22 | -8275.982246 | 0.07119 0.19579 | None |
|  | free ratio | 39 | -8264.830402 | 0.11686 0.10438 0.11293 0.06790 0.26615 0.05903 0.08463 0.05235 0.03788 0.08486 0.09992 0.09315 0.09086 0.06426 0.26649 0.07367 0.03596 0.03981 0.08612 | None |
| Site model | M0 | 21 | -8276.691385 | 0.0727 | None |
|  | M1a | 22 | -8101.888629 | p: 0.86855 0.13145  w: 0.04057 1.00000 | None |
|  | M2a | 24 | -8101.888642 | p: 0.86855 0.13145 0.00000  w: 0.04057 1.00000 48.15144 | None |
|  | M3 | 25 | -8011.407980 | p: 0.64198 0.27634 0.08168  w: 0.00563 0.15592 0.63794 | None |
|  | M7 | 22 | -8012.532145 | p = 0.19564 q = 1.69171 | None |
|  | M8 | 24 | -8012.284911 | p0 =0.97790 p =0.21619 q =2.28865  (p1 =0.02210) w =1.00000 | None |
| Branch-site model | M0 | 23 | -8101.798614 | site class 0 1 2a 2b  proportion 0.85182 0.12884 0.01680 0.00254  background w 0.04027 1.00000 0.04027 1.00000  foreground w 0.04027 1.00000 1.00000 1.00000 | None |
|  | MA | 24 | -8101.798614 | site class 0 1 2a 2b  proportion 0.85182 0.12884 0.01680 0.00254  background w 0.04027 1.00000 0.04027 1.00000  foreground w 0.04027 1.00000 1.00000 1.00000 | None |
