# Supplementary material for: Genome-wide identification, characterization and expression analysis of the BMP family associated with beak-like teeth in Oplegnathus
Source: Front Genet. 2022 Jul 18;13:938473. doi: 10.3389/fgene.2022.938473 (PMC9342863; doi:10.3389/fgene.2022.938473)
Supplement: Supplementary file 1 [file DataSheet1.ZIP › Table S9. BMP6 model parameter estimates and log-likelihoods.docx]

Table S9. BMP6 model parameter estimates and log-likelihoods

|  | Model | np | lnL | omega | Positive selection  site(BEB) |
| --- | --- | --- | --- | --- | --- |
| Branch model | one ratio | 23 | -9356.611173 | 0.0938 | None |
|  | two ratio | 24 | -9356.033137 | 0.09497 0.04001 | None |
|  | free ratio | 43 | -9309.575809 | 0.02454 0.02484 999.00000 0.08430 0.40259 0.03602 0.09474 0.08122 0.13640 0.26903 0.17243 0.09306 147.64841 161.20860 0.06064 0.13161 0.05076 0.08183 0.07033 0.04976 0.11042 | None |
| Site model | M0 | 23 | -9356.611173 | 0.0938 | None |
|  | M1a | 24 | -9184.648124 | p: 0.86663 0.13337  w: 0.06413 1.00000 | None |
|  | M2a | 26 | -9184.648124 | p: 0.86663 0.00365 0.12971  w: 0.06413 1.00000 1.00000 | None |
|  | M3 | 27 | -9108.706334 | p: 0.49581 0.39227 0.11192  w: 0.01264 0.13818 0.64941 | None |
|  | M7 | 24 | -9114.820334 | p = 0.38417 q = 2.51040 | None |
|  | M8 | 26 | -9111.588630 | p0 =0.94979 p =0.48592 q =4.35925  (p1 =0.05021) w =1.00000 | None |
| Branch-site model | M0 | 25 | -9184.648124 | site class 0 1 2a 2b  proportion 0.86663 0.13337 0.00000 0.00000  background w 0.06413 1.00000 0.06413 1.00000  foreground w 0.06413 1.00000 1.00000 1.00000 | None |
|  | MA | 26 | -9186.731571 | site clas 0 1 2a 2b  proportion 0.00005 0.00001 0.86770 0.13225  background w 0.06368 1.00000 0.06368 1.00000  foreground w 0.06368 1.00000 1.00000 1.00000 | None |
