# Supplementary figures and images for: Genome-wide identification, characterization and expression analysis of the BMP family associated with beak-like teeth in Oplegnathus
Source: Front Genet. 2022 Jul 18;13:938473. doi: 10.3389/fgene.2022.938473 (PMC9342863; doi:10.3389/fgene.2022.938473)

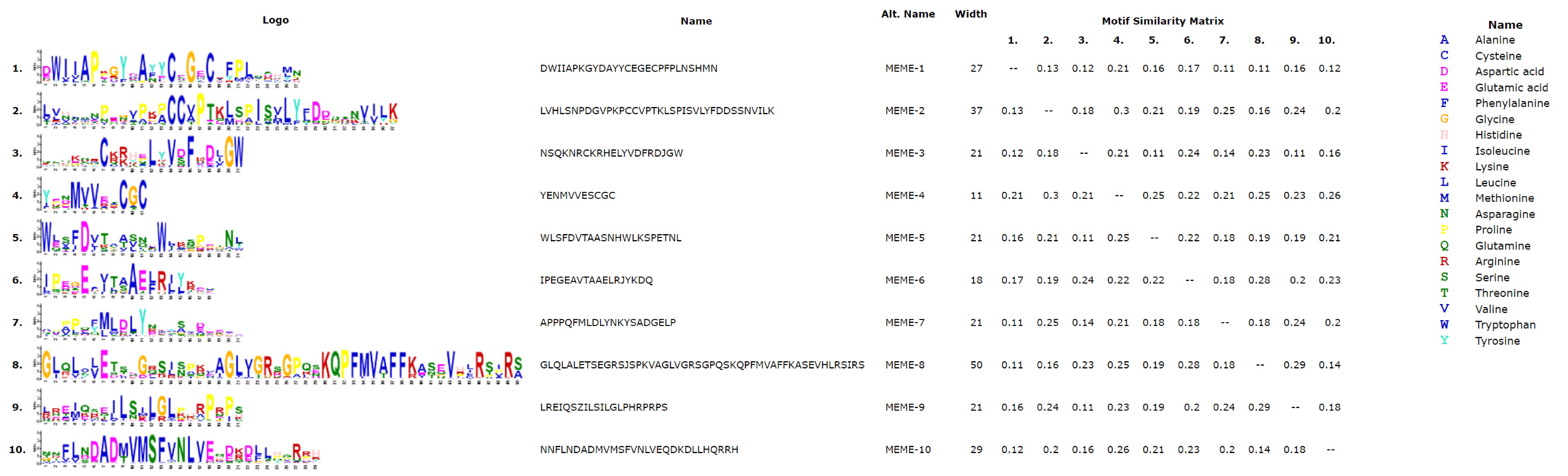

Supplement: Supplementary file 1 [file DataSheet1.ZIP › Figure S1.The motif of the BMP protein in Oplegnathus and T. rubripes.jpg]
